# The Biophysical Basis Underlying Gating Changes in the p.V1316A Mutant Na_v_1.7 Channel and the Molecular Pathogenesis of Inherited Erythromelalgia

**DOI:** 10.1371/journal.pbio.1002561

**Published:** 2016-09-21

**Authors:** Chiung-Wei Huang, Hsing-Jung Lai, Po-Yuan Huang, Ming-Jen Lee, Chung-Chin Kuo

**Affiliations:** 1 Department of Physiology, National Taiwan University College of Medicine, Taipei, Taiwan; 2 Department of Neurology, National Taiwan University Hospital Jinshan Branch, New Taipei City, Taiwan; 3 Department of Neurology, National Taiwan University Hospital, Taipei, Taiwan; University of Zurich, SWITZERLAND

## Abstract

The Na_v_1.7 channel critically contributes to the excitability of sensory neurons, and gain-of-function mutations of this channel have been shown to cause inherited erythromelalgia (IEM) with neuropathic pain. In this study, we report a case of a severe phenotype of IEM caused by p.V1316A mutation in the Na_v_1.7 channel. Mechanistically, we first demonstrate that the Na_v_β4 peptide acts as a gating modifier rather than an open channel blocker competing with the inactivating peptide to give rise to resurgent currents in the Na_v_1.7 channel. Moreover, there are two distinct open and two corresponding fast inactivated states in the genesis of resurgent Na^+^ currents. One is responsible for the resurgent route and practically existent only in the presence of Na_v_β4 peptide, whereas the other is responsible for the “silent” route of recovery from inactivation. In this regard, the p.V1316A mutation makes hyperpolarization shift in the activation curve, and depolarization shift in the inactivation curve, vividly uncoupling inactivation from activation. In terms of molecular gating operation, the most important changes caused by the p.V1316A mutation are both acceleration of the transition from the inactivated states to the activated states and deceleration of the reverse transition, resulting in much larger sustained as well as resurgent Na^+^ currents. In summary, the genesis of the resurgent currents in the Na_v_1.7 channel is ascribable to the transient existence of a distinct and novel open state promoted by the Na_v_β4 peptide. In addition, S4–5 linker in domain III where V1316 is located seems to play a critical role in activation–inactivation coupling, chiefly via direct modulation of the transitional kinetics between the open and the inactivated states. The sustained and resurgent Na^+^ currents may therefore be correlatively enhanced by specific mutations involving this linker and relevant regions, and thus marked hyperexcitability in corresponding neural tissues as well as IEM symptomatology.

## Abstract 2

Mutations in the Na_v_1.7 sodium channel cause idiopathic erythromelalgia. This study shows that the pathogenic resurgent sodium currents arise via modification of gating behavior rather than via competing pore block by the Na_v_β4 peptide.

## Author Summary

The gain-of-function mutation (p.V1316A) of the Na_v_1.7 channel causes inherited erythromelalgia (IEM), a disease characterized by extremely enhanced activity in relevant neural tissues that results in neuropathic pain. We found that the p.V1316A mutation alters the basic gating properties of the channel, leading to increased sustained currents during membrane depolarization and resurgent currents during repolarization. Neurons expressing these mutant channels are more difficult to maintain in a hyperpolarized state and are thus more excitable. We demonstrate that there is very likely a distinct set of open/inactivated (O/I) states responsible for the genesis of resurgent currents. We show that the p.V1316A mutation chiefly accelerates the I to O transition in this set, but also decelerates the transitions between different sets of O/I states, to cause the channel gating and cellular excitability changes. Contrary to the conventional view, we find that the Na_v_β4 peptide, a key element responsible for sizable resurgent currents, does not seem to act as a pore blocker that competes with the inactivation peptide. Instead, we show that it acts as a gating modifier of the Na_v_1.7 channel. Thus, the DIII/S4–5 linker, where V1316 is located, may play a critical role not only in O/I coupling but also in the couplings between different sets of O/I in the Na_v_1.7 channel.

## Introduction

Voltage-gated sodium channels, such as the Na_v_1.7 channel (~1,980 amino acids; ~225 kDa), are heteromeric protein complexes composed chiefly of a pore loop-forming alpha subunit, which is a large transmembrane protein containing four similar domains, and an auxiliary beta subunit [1–3]. The Na_v_1.7 channel is abundantly expressed in the neurons of trigeminal, sympathetic, and dorsal root ganglia (DRG) [2,4,5]. Mutations in the Na_v_1.7 channel may result in severe disorders involving the peripheral nervous system such as paroxysmal extreme pain disorder (PEPD, OMIM 167400), congenital insensitivity to pain (CIP, OMIM 243000), and inherited erythromelalgia (IEM, OMIM 133020) [6–13]. Recently, two sporadic cases of the p.V1316A (located in the DIII/S4–5 linker) mutation were reported [14,15], demonstrating a very severe form of IEM, which is characterized by extremely enhanced activity in relevant neural tissues [16–18]. It would be desirable to explore the molecular and biophysical basis how this mutant Na_v_1.7 channels could be responsible for such exteme neural activities.

Structurally, each alpha subunit of the Na_v_1.7 channel is composed of four homologous domains (D1–D4) and with six transmembrane segments (S1–S6) in each domain [1–3]. The S4 segment acts as a voltage sensor while the loop between S5 and S6 segments lines at the external of the pore containing the selectivity filter [3,19]. Upon depolarization, the S4 voltage sensor moves outward to pull on the S4–S5 linker in each domain, and then on the bundle-crossing region of the S6 segment (the “S6 gate”) to open the channel gate [1–3]. IEM and PEPD are both characterized by episodes of severe pain and “gain-of-function” mutations of the Na_v_1.7 channel [8,9,12,20], although IEM more likely involves extremities than rectal or ocular areas in clinical considerations. Previous studies have shown that quite a few IEM-causing mutations are located in the S4–S5 linker in each domain, such as p.I234T, p.I848T, p.G856D, p.L858F, p.L858H, p.P1308L, and p.V1316A mutations [11]. Accordingly, these mutations have been reported to induce quite a few gating changes including depolarization shift of the activation curve, slowing of slow deactivation time constants, and increase of the response to slow depolarization of ramp currents [14,15,21–24]. Recently, the T1464I (in D3–D4 linker), M1627K (in S4–S5 linker /D4), and p.A1632E (in S4–S5 linker/D4) mutant channels showed enhanced resurgent Na^+^ currents [25,26], which may play a critical role in the genesis of repetitive or burst neuronal discharges [25]. Conventionally, the genesis of resurgent currents has been ascribed to direct competition between the Na_v_β4 peptide and the inactivating peptides for the same open state of the channel, and consequently re-opening of the Na_v_β4-blocked but not the inactivated channel during the repolarization phase just following a depolarization [27–30]. Why and how the resurgent currents are enhanced by these point mutations, however, have remained unexplored. We investigated the changes in molecular behavior of the p.V1316A mutant channel which causes the severe clinical IEM symptomatology. We found that the Na_v_β4 peptide acts as a gating modifier rather than an open channel blocker to generate resurgent Na^+^ currents. There is a novel open state that is responsible for the genesis of resurgent current and is significantly existent only in the presence of Na_v_β4 peptide. The p.V1316A mutation markedly increases both the sustained and the resurgent Na^+^ currents, mostly ascribable to the destabilized inactivated states by both acceleration of the transition from the inactivated to the open states and deceleration of the reverse transition. These findings not only account well for the molecular mechanism underlying the most severe clinical presentations of IEM but also strongly implicate that S4–5 linker/D3, where V1316 is located, plays a critical role in the molecular operations of recovery from fast inactivation and thus genesis of resurgent currents in Na^+^ channels.

## Results

### Clinical Features of the Index Patient

**Fig 1:**
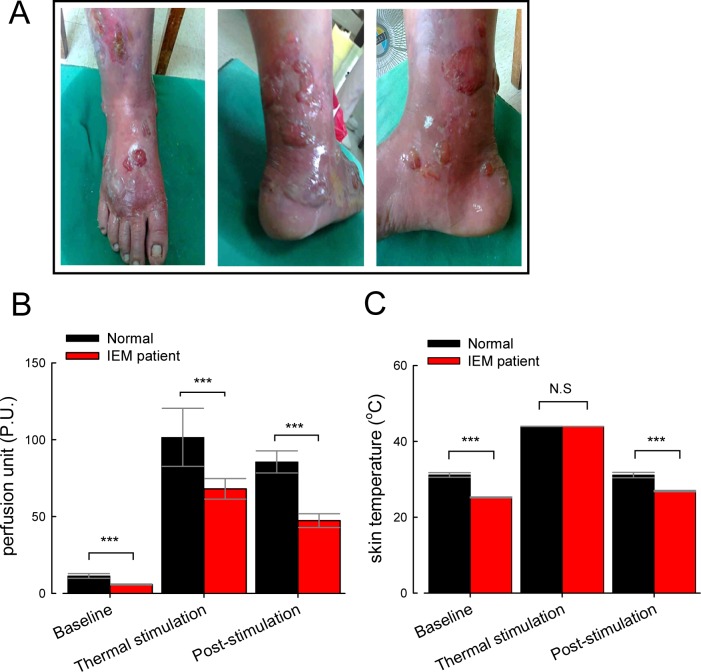
Clinical features of the index patient with erythromelalgia (IEM). (A) Large areas of swelling erythema with blisters and wounds in the patient’s right foot. (B) Perfusion unit (P.U.) of the IEM patient (six measurements) and normal controls (six subjects, four measurements for each) at baseline, thermal stimulation (up to 44°C in 1 min) and 10 min cooling after thermal stimulation (“post-stimulation”) (***, *p* < 0.001). (C) Skin temperature of the right foot at baseline, thermal stimulation, and post-stimulation (***, *p* < 0.001; N.S., no statistically significant difference). Individual data is shown in the file of S1 Data.

An 18-yr-old Taiwanese girl suffered from severe burning pain and reddish erythema on both feet (Fig 1A) with the diagnosis of primary erythromelalgia [15]. Genetic analysis showed a missense mutation (p.V1316A) of human Na_v_1.7 (*SCN9A*) gene. The excruciating pain made her immerse her feet into ice-cold water from time to time. The local skin infection was associated with lesions, such as blisters, and the wound healing was poor despite intensive antibiotic therapy and local surgical debridement. Laser Doppler study revealed a profound reduction in the perfusion unit (P.U.) of skin capillary in the feet at resting state (Fig 1B). The phenomenon was also found at both thermal stimulation and post-stimulation state, indicative of a profound reduction of capillary perfusion in the index patient even in the remission or “pain-free” state. In line with the perfusion study, the baseline skin temperature of the patient was markedly lower than normal control (Fig 1C). The skin temperature reached roughly the same level for both the patient and control subjects during thermal stimulation (44°C). However, the patient’s skin temperature was once again significantly lower than that of the control’s post cooling at room temperature for 10 min (Fig 1C). These findings strongly implicate markedly enhanced sympathetic activities associated with IEM. Clinically, infection and inflammation very much aggravated the neuropathic pain. Unfortunately, she developed bouts of extreme pain followed by hypotension and shock with subsequent mortality despite exhaustive resuscitation.

### Similar Decay Time Constants of Transient Currents but Distinct Genesis of Resurgent Currents of the wild-type (WT) Na_v_1.7 Channel in the Presence and Absence of the Na_v_β4 Peptide

**Fig 2:**
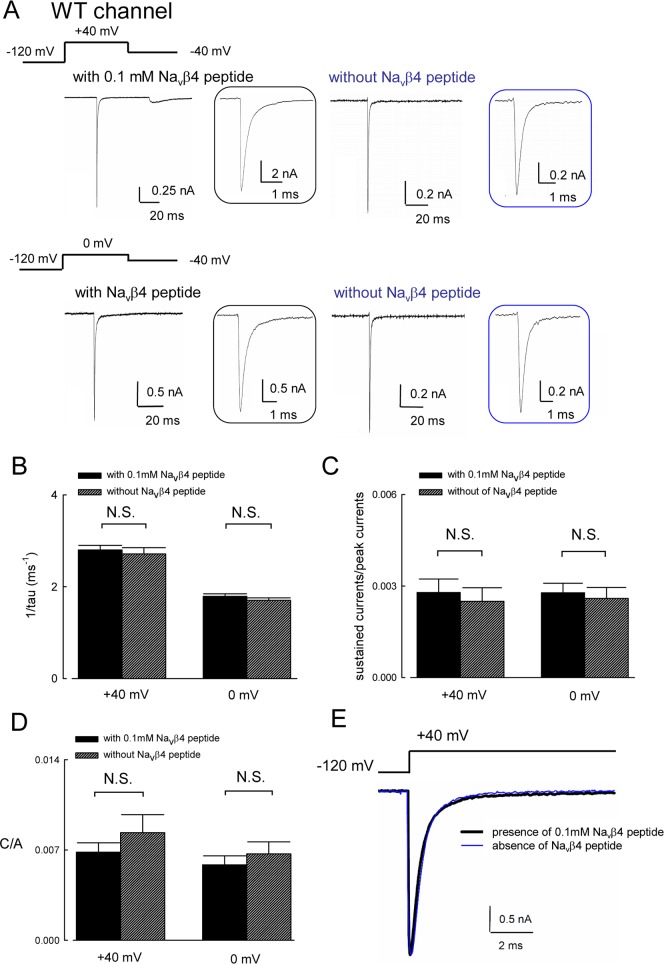
Resurgent and transient Na^+^ currents of WT Na_v_1.7 channel in the presence and absence of the Na_v_β4 peptide. (A) The cells were held at –120 mV, and the resurgent Na^+^ currents were evoked by a voltage steps to –40 mV after a prepulse to either +40 mV or 0 mV for 30 ms in the presence or absence of 0.1 mM Na_v_β4 peptide. Inset figures: the transient currents during the prepulses are magnified in the time axis. (B) The decay phase of the transient currents from the 95% of the peak to the steady-state current was fitted by a standard exponential function: *f*(x) = *A*×exp(–t/*τ*)+ C. Note that the time constants of the decay phase of transient Na^+^ currents at +40 mV prepulse are shorter than that at 0 mV, but both remain the same in the presence and absence of 0.1 mM Na_v_β4 peptide (*n* = 15; N.S., no statistically significant difference). (C) Cumulative results were obtained from experiments with similar protocols described in part **A** but with longer prepulses to either +40 or 0 mV for ~100 ms in the WT channel. The sustained currents are defined as the average currents from 75 to 80 ms after peak transient current (and normalized to the peak transient current in the same sweep). At either +40 or 0 mV depolarization, there is no significant difference in the normalized sustained currents in the presence and absence of 0.1 mM Na_v_β4 peptide (*n* = 15; N.S., no statistically significant difference). (D) Cumulative results of C/A values from part B (C is divided by A from the same fit) are also compared to further substantiate the unaltered sustained currents by the Navb4 peptide either at +40 or 0 mV (*n* = 15 for each condition; N.S., no statistically significant difference). (E) The transient Na^+^ currents are adjusted to the same peak current amplitude and then average currents are obtained in the presence and absence of 0.1 mM Na_v_β4 peptide (*n* = 15 for each condition). Note that the two average currents are essentially superimposable. Individual data is shown in the file of S1 Data.

**Fig 3:**
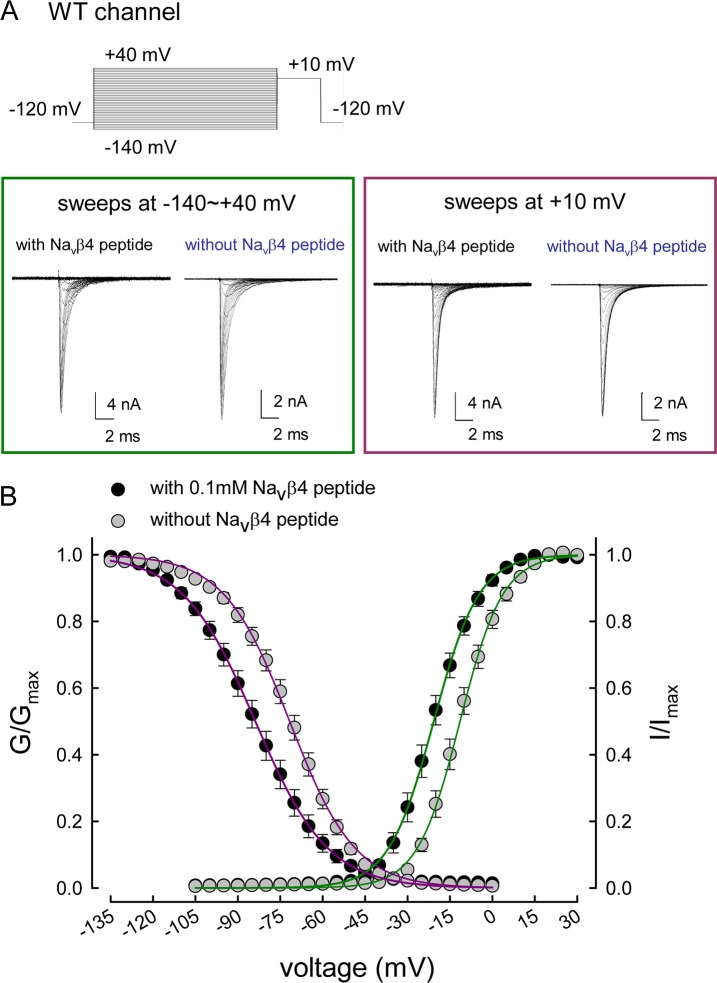
The activation and inactivation curves of the WT Na_v_1.7 channel in the presence and absence of the Na_v_β4 peptide. (A) Sample sweeps for the making of the activation (green box, on the left) and inactivation (purple box, on the right) curves in the presence and absence of the Na_v_β4 peptide are shown (see Materials and Methods for the experimental protocols). (B) The activation and inactivation curves for the each cell are fitted with Boltzmann functions, and the cumulative results for V_h_ are –20.74±1.47 mV and –11.19±1.21 mV with and without the Na_v_β4 peptide, respectively (*n* = 15; *p* < 0.05), and *k* are 7.11±0.33 and 6.90±0.41 with and without the Na_v_β4 peptide fort the inactivation curve, respectively (*n* = 15; no significant difference). For the inactivation curves, the V_h_ are –83.64±2.17 mV and –71.80±1.61 mV with and without the Na_v_β4 peptide, respectively (*n* = 15; *p* < 0.05), and *k* are –11.55±0.43 and –10.89±0.37 with and without the Na_v_β4 peptide, respectively (*n* = 15; no significant difference). Single Boltzmann functions fits to the mean values are for the activation curve, the V_h_ and *k* are ~–20.7 mV and ~8.0 with 0.1 mM Na_v_β4 peptide, and ~–11.43 mV and ~7.6 without the Na_v_β4 peptide, respectively; for the inactivation curves, the V_h_ and *k* are ~–83.8 mV and ~12.8 with 0.1 mM Na_v_β4 peptide and ~–71.7 mV and ~11.6 without the Na_v_β4 peptide, respectively. Individual data is shown in the file of S1 Data.

To investigate the molecular functional changes of the p.V1316A mutant Na_v_1.7 channel, we first characterized the basic key biophysical properties of the WT channel. Fig 2A shows that the resurgent Na^+^ currents are present only in the presence, but not in the absence of the Na_v_β4 peptide when there is a prepulse at +40 mV. In contrast, there are much smaller or little resurgent Na^+^ currents with a prepulse at 0 mV, either in the presence or in the absence of 0.1 mM Na_v_β4 peptide. It has been proposed that the Na_v_β4 competes with the fast inactivating peptide to generate resurgent currents [27,28,31,32]. If the Na_v_β4 peptide is indeed an open channel blocker acting fast enough to compete with fast inactivation (and thus keeping the channel in an open but non-conducting state responsible for the genesis of resurgent currents upon subsequent repolarization), then the presence of Na_v_β4 peptide should accelerate the decay of the transient Na^+^ currents at least at the +40 mV step depolarization (prepulse), where prominent resurgent currents are generated in the following repolarization phase. The cumulative results in Fig 2B show that the decay time constants of the transient Na^+^ currents in the WT channel remain essentially the same in either the presence or absence of the Na_v_β4 peptide at both +40 and 0 mV prepulse. These findings strongly implicate that the genesis of resurgent Na^+^ currents in the presence of the Na_v_β4 peptide cannot be explained simply with the conventional model inferring the competition between the open channel blockers Na_v_β4 peptide and the inactivating peptides, and that the open state responsible for the resurgent currents probably is not the same as that giving rise to the transient Na^+^ currents in the depolarization prepulse. Fig 2C and 2D further shows that the relative sustained Na^+^ currents (the ratio between sustained and peak of transient Na^+^ currents) also remain the same whether the Na_v_β4 peptide is present or not. The unaltered sustained and decay kinetics of transient Na^+^ currents are further substantiated by the essentially superimposable average Na^+^ currents in the presence and absence of 0.1 mM Na_v_β4 peptide (Fig 2E). These findings once more argue against the open channel blocked by Na_v_β4 peptide and are consistent with the transient nature of the gating states responsible for the genesis of resurgent currents (see Discussion). We therefore explored the role of the Na_v_β4 peptide as a gating modifier in addition to the open channel pore-blocking effect. Fig 3A and 3B shows that both the activation and inactivation curves of the WT channel are evidently shifted leftward (~12 mV) on the voltage axis in the presence of the Na_v_β4 peptide. The slope of the curves, however, is essentially unchanged. These results once again support the role of Na_v_β4 peptide as a gating modifier, which does not alter the voltage dependence of WT channel opening and inactivation but changes the non-electric free energy difference between the deactivated (closed) and the activated conformations by a fixed amount.

### Larger Sustained Na^+^ Currents in the p.V1316A Mutant than in the WT Channel

**Fig 4:**
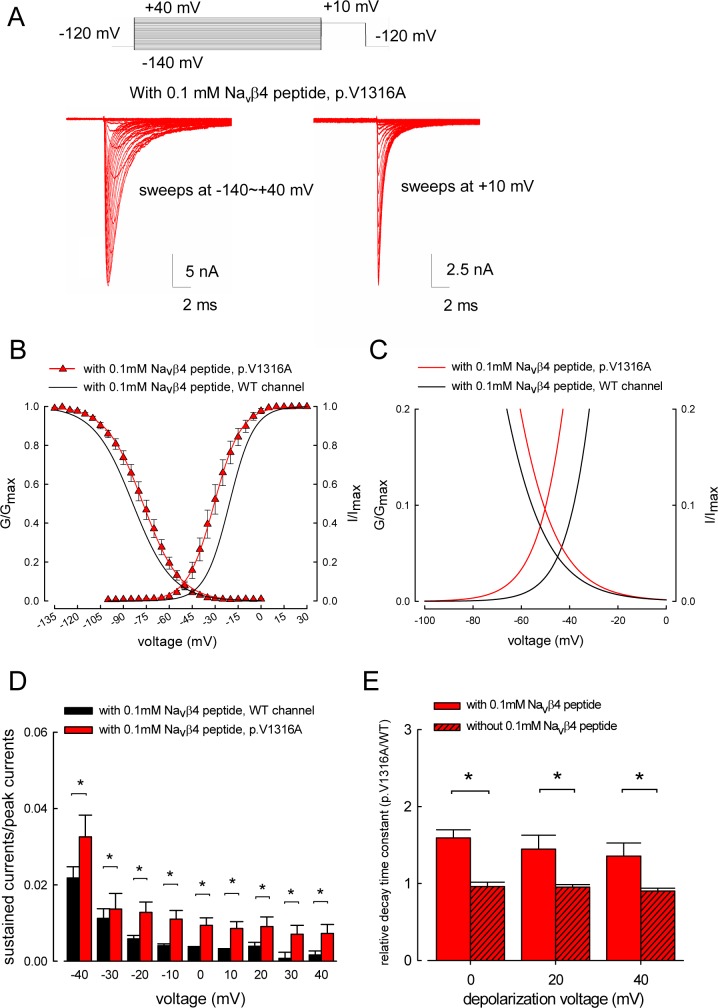
The activation and inactivation curves of the p.V1316A mutant channel in the presence of the Na_v_β4 peptide. (A) Sample sweeps for the making of the activation (left panel, currents during the –140 to +40 mV prepulse) and inactivation (right panel, currents during the +10 mV pulse following the prepulse) curves of the p.V1316A mutant channel in the presence of the Na_v_β4 peptide are shown (see Materials and Methods for the experimental protocols). (B) The activation and inactivation curves for each cell are also fitted with Boltzmann functions, and the cumulative results for V_h_ and *k* are –30.48±2.45 mV (*n* = 12; *p* < 0.05, versus WT channel) and 8.05±0.51 (*n* = 12; no significant difference versus WT channel) for the activation curves, respectively. For the inactivation curves, V_h_ and *k* are –77.04±2.17 mV (*n* = 12; *p* < 0.05, versus WT channel) and –10.9±0.39 (*n* = 12; no significant difference versus WT channel) in the p.V1316A mutant channel, respectively. Single Boltzmann fits to the mean values are for the p.V1316A mutant channel: V_h_ and *k* are ~–30.7 mV and ~8.7; for the activation curve; V_h_ and *k* are ~–77.1 mV and ~12.0 for the inactivation curve. The solid black lines for the WT channel are taken from Fig 2B for comparison. (C) A closer view of the fitting lines in part **B** between –100 and 0 mV. (D) Cumulative results were obtained with the same protocols described in part **A** for the WT and p.V1316A mutant channels (*n* = 15 for the WT channels; *n* = 12 for the p.V1316A mutant channels). The ratio between the sustained and peak transient currents (in the same sweep) is significantly larger in the p.V1316A mutant channel with depolarization potentials between –40 and +40 mV. *, *p* < 0.05. (E) At each depolarization voltage (0, +20, and +40 mV), the time constants (tau) of the decay (inactivation) phase of transient Na^+^ currents in the p.V1316A mutant channel are normalized to the average time constants of the inactivation phase of transient Na^+^ currents in the WT channel to give the relative inactivation tau, either in the presence or absence of 0.1 mM Na_v_β4 peptide. The cumulative results (*n* = 10–12 for each measurement) show that the relative inactivation time constant at each depolarization voltage is significantly lengthened by the p.V1316A mutation only in the presence but not in the absence of the Na_v_β4 peptide. *, *p* < 0.05. Individual data is shown in the file of S1 Data.

Fig 4 compares the basic gating behaviors of the p.V1316A mutant with the WT Na_v_1.7 channel. The activation and inactivation curves of p.V1316A mutant channel are negatively and positively shifted in the voltage axis, respectively. On the other hand, the slope of both gating curves is again grossly unchanged or only minimally changed by the mutation (Fig 4B). These results indicate that p.V1316A mutant channel can be more activated but less inactivated especially at a specific range of membrane potentials. Fig 4C shows the presumable range of this predicted “window” or sustained current, which could be defined by the area under both the activation and inactivation curves. Consistently, the increase in sustained currents in the p.V1316A mutant channel could be demonstrated with direct measurement of the late currents during depolarizing pulse (Fig 4D), and may therefore account for part of the origin of nerve hyperexcitability in IEM. In theory, the sustained Na^+^ currents may signal a decrease in the inactivation rate (slowed transition from the open to the inactivation states) or an increase in the reverse rate (accelerated transition from the inactivated to the open state) or both. Fig 4E shows that in the p.V1316A mutant channel, the decay of transient currents (the macroscopic inactivation rate) is 50% slowed by the Na_v_β4 peptide. On the other hand, only in the presence of the Na_v_β4 peptide, not in the absence of the Na_v_β4 peptide, the macroscopic inactivation rates are ~50% slowed by the p.V1316A mutation. In other words, the Na_v_β4 peptide slows rather than accelerates decay of transient currents in the p.V1316A mutant channel, and the p.V1316A mutation slows the macroscopic inactivation rates of the channel only in the presence but not in the absence of the Na_v_β4 peptide, although the Na_v_β4 peptide by itself has no apparent effect on the inactivation kinetics of the WT channel (Fig 1B). These findings further substantiate that both the Na_v_β4 peptide and the p.V1316A mutation are gating modifiers of the Na_v_1.7 channel, and p.V1316A mutation may at least decrease the inactivation rate of the channel.

### Peak Resurgent Na^+^ Currents at –40~–60 mV but Monotonous Acceleration of Current Decay by Membrane Hyperpolarization

**Fig 5:**
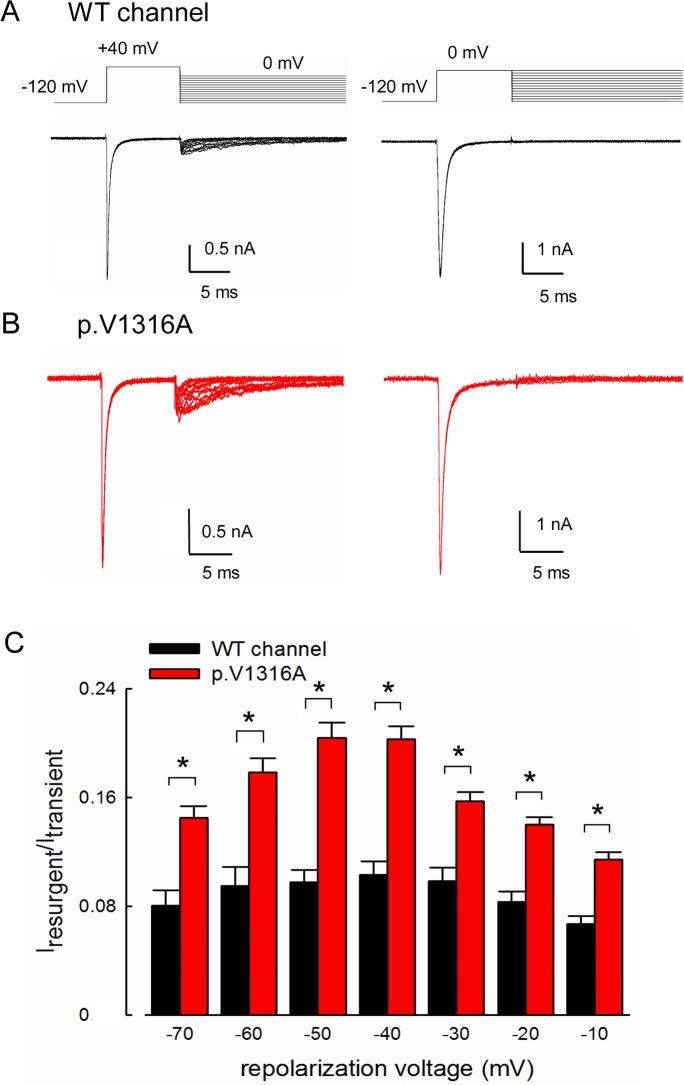
Larger resurgent Na^+^ currents in the p.V1316A mutant than that in the WT channels. (A) The cells were held at –120 mV, and the resurgent Na^+^ currents of WT channel were evoked by pulses between 0 and –120 mV in 10 mV increments following a depolarization prepulse of either +40 mV or 0 mV for 10 ms in the presence of the Na_v_β4 peptide. (B) Sample sweeps for the p.V1316A mutant channel in the presence of 0.1 mM Na_v_β4 peptide following the same protocol as part **A.** (C) Cumulative results were obtained from experiments described in part **A** for the WT and p.V1316A mutant channels (each *n* = 10). The ratio between resurgent and peak transient Na^+^ current (in the same sweep) is significantly larger in the p.V1316A mutant than WT channels at repolarization potentials between –10 and –70 mV. *, *p* < 0.05. Individual data is shown in the file of S1 Data.

**Fig 6:**
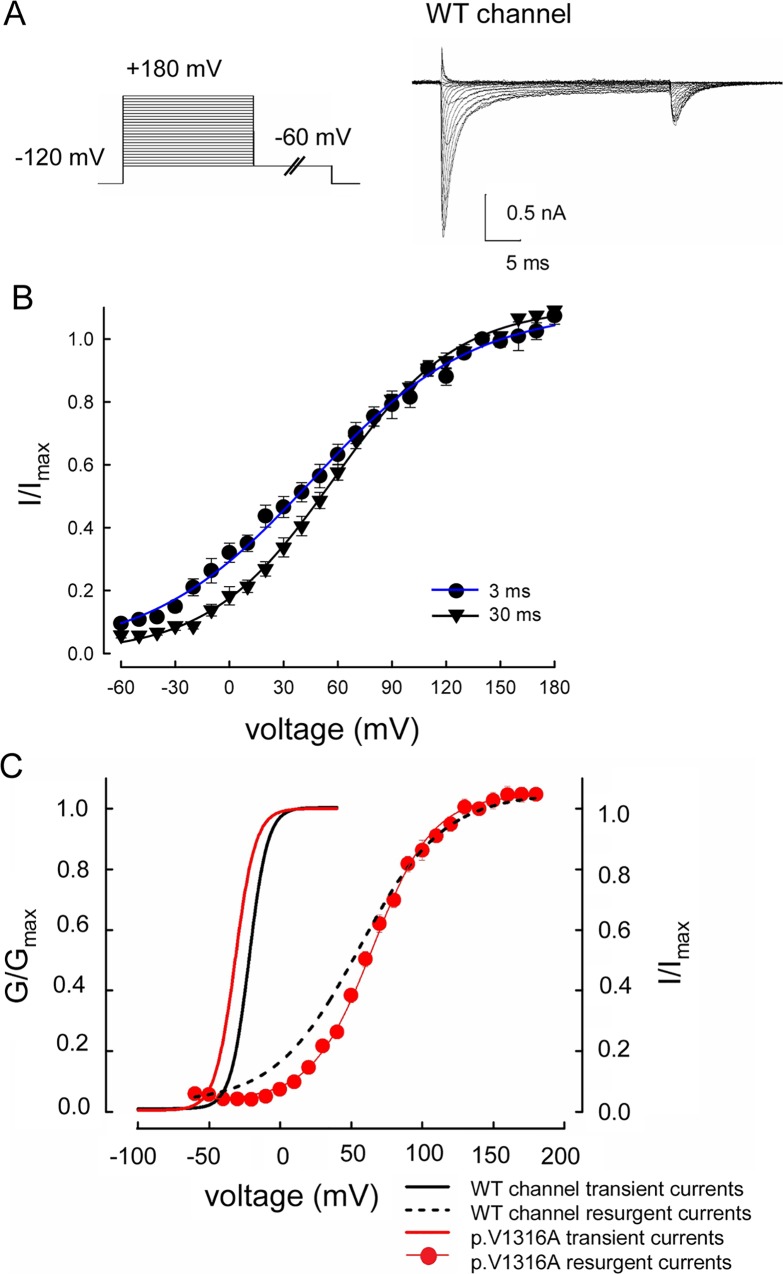
The activation curve of resurgent Na^+^ currents in the WT and p.V1316A mutant channels. (A) Sample sweeps were obtained in the presence of 0.1 mM Na_v_β4 peptide for the WT channel. The cell was first held at –120 mV for ~30 ms, and then stepped to different depolarization prepulses between –60 and +180 mV for 30 ms in 10 mV increment. Resurgent Na^+^ currents were then evoked by a pulse to –60 mV for 150 ms. (B) The relative magnitude of resurgent Na^+^ currents for the WT channel is defined by normalization of the peak amplitude of resurgent Na^+^ currents at –60 mV following different prepulses to that following a prepulse of +140 mV in the same cell, and then plotted against the prepulse potential. The same experiments were also repeated with prepulses only 3 ms in length. The resurgent activation curve for each cell are also obtained by fittings with Boltzmann functions, and the cumulative results for V_h_ are 38.63±4.39 mV and 49.19±2.58 mV for 3 ms and 30 ms prepulses, respectively (*n* = 10 for the 3 ms; *n* = 9 for the 30 ms), and *k* are 39.40±3.37 and 29.01±1.46 for 3 ms and 30 ms prepulses, respectively. Single Boltzmann fits to the mean values are for 3 ms: V_h_ = ~44.93 mV, *k* = ~43.63, and for 30 ms: V_h_ = ~48.63 mV, *k* = ~28.59. (C) The activation curve of resurgent Na^+^ currents in the p.V1316A mutant channel is obtained with the same protocols as that in part **B** (prepulse = 30 ms, *n* = 10). The activation curves of transient Na^+^ currents for the WT and p.V1316A mutant channels and the activation curves of resurgent Na^+^ currents in the WT channels (the fitting lines in Figs 3B and 4B, and part **B**, respectively) are replotted for comparison. The resurgent activation curve of p.V1316A mutant channels for each cells are also obtained by fitting Boltzmann functions, and the cumulative results for V_h_ and *k* are 59.76±2.17 mV (*n* = 10; *p* < 0.05 versus the activation curve of transient currents in the p.V1316A mutant channels) and 21.70±1.14 (*n* = 10; *p* < 0.05 versus the activation curve of transient currents in the p.V1316A mutant channels), respectively. Single Boltzmann fits to the mean values are for p.V1316A mutant channel: V_h_ = ~59.5 mV and *k* = ~21.39. Note that the activation curves of resurgent Na^+^ currents in both WT and p.V1316A mutant channels are markedly shifted to the more positive voltage range and with a much less steep slope than those for the transient currents. Individual data is shown in the file of S1 Data.

Resurgent Na^+^ currents are only discernible in the presence of Na_v_β4 peptide, which is more likely a gating modifier than a fast open channel blocker (Figs 2 and 3). The voltage dependence of the kinetics and amplitude of the resurgent current were thus further assessed. Fig 5 shows that prominent resurgent currents are elicited at 0~–120 mV following a prepulse at depolarization of +40 mV but not 0 mV in both WT and p.V1316A mutant channels, again demonstrating the requirement of much more positive potentials to elicit resurgent than transient currents (see also Fig 6C below). We then investigated the possibility of accelerated transitions from the inactivated to the open states, which would also increase the resurgent in addition to the sustained Na^+^ currents (see Discussion). Fig 5C shows that the resurgent currents indeed are significantly larger in the p.V1316A mutant channel than in the WT channel in the presence of the Na_v_β4 peptide. These results suggest that DIII/S4–5 linker, where p.V1316A mutation is located, may play a critical role in the genesis of resurgent currents by modulation of the kinetics of transitions between the correlative inactivated and open states of the channel in both directions. These altered gating properties by the p.V1316A mutation could be in turn responsible for the extremely heightened neural activities and clinical manifestations of IEM.

### Distinct Activation Curves of Transient and Resurgent Na^+^ Currents in the WT Channel

We then investigated the voltage-dependent occupation of the open state that gives rise to the resurgent Na^+^ currents. Depolarization prepulses with 3 and 30 ms yield similar “activation curves” of the resurgent currents (Fig 6A and 6B), although the 3 ms prepulses tend to result in higher proportion of the open state toward the more negative voltages (see below), suggesting that most of the open state responsible for the resurgent currents is well accessible with a 3 ms depolarization. The most striking features of the activation of the resurgent currents are the much shallower voltage dependence but a marked positive shift in the voltage axis if compared to the activation curves of the transient currents (Fig 6C). This is not compatible with the evident negative shift of the activation/inactivation curves by Na_v_β4 peptide (Fig 3B) based on the conventional model. If the resurgent particle or Na_v_β4 could effectively bind to the open channel pore only upon that strong depolarizing prepulses and thus gives rise to resurgent current at the following repolarization phase, then the activation/inactivation curves which are located at a more negative range in the voltage axis should not be effectively shifted (especially shifted to more negative potentials). On the other hand, the activation curve of resurgent currents in the p.V1316A mutant channel is very similar to or even slightly positively shifted than that in the WT channel. Given the significant increase of both sustained and resurgent currents by the p.V1316A mutation (Figs 4D and 5C), this finding is again difficult to envisage with the conventional model. The competition between resurgent and inactivating particles should become more favorable for the resurgent particle because of significantly decreased tendency of inactivating particle binding by the mutation (so that the resurgent and sustained current could be both increased). The relatively weakened competition of inactivating particle more likely should shift the activation curve of resurgent currents negatively (rather than unchanged or even shifted positively). The characteristics of the activation curve of the resurgent currents are thus not in accordance with the conventional pore-block model, but much more consistent with the proposed new two-open-state or gating modification model. These findings not only lend strong support to a new open state responsible for the resurgent currents, but also suggest a distinct position of the gating voltage sensors of the “resurgent” open state from the “conventional” one (see Discussion).

### Slower Time to Peak but Unchanged Decay Kinetics of the Resurgent Currents in the p.V1316A Mutant Channel

**Fig 7:**
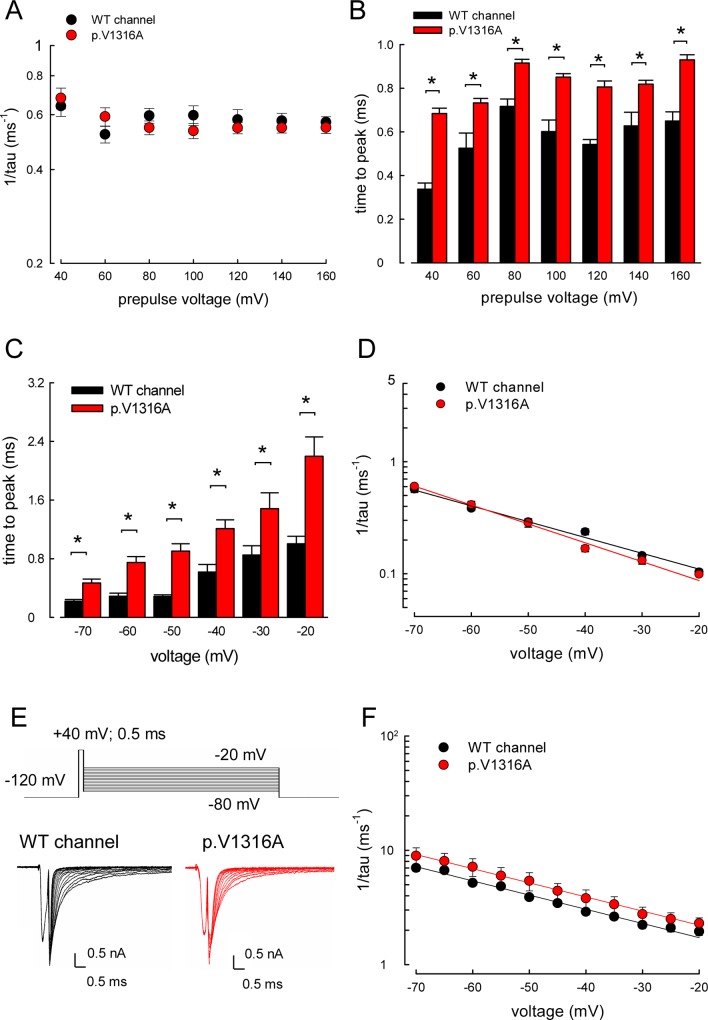
Monotonously accelerated decay of resurgent Na^+^ currents by membrane hyperpolarization in the WT and p.V1316A mutant channels. (A) Cumulative results were obtained with the same protocols described in Fig 6A (each *n* = 10). The inverses of decay time constants of the resurgent Na^+^ currents are obtained at –60 mV after prepulses of +40~+160 mV for ~10 ms in 20 mV increment for WT and p.V1316A mutant channels. Note that the inverse decay time constants are very similar in WT and p.V1316A mutant channels. (B) Cumulative results were obtained from the experiments described in part **A** (each *n* = 10). Note that the time to peak of the resurgent Na^+^ currents in the p.V1316A mutant channel is always slower than that in the WT channel at all of the prepulse voltages tested. *; *p* < 0.05. (C) The time to the resurgent Na^+^ current peak (the time from ending of depolarization prepulse of +40 mV to the resurgent current peak at different voltages) was measured with the same protocol in Fig 5 and plotted against the voltage of the resurgent pulse in the WT and p.V1316A mutant channels (each *n* = 10). Note trend of voltage dependence. Note that the time to the resurgent Na^+^ currents peak is evidently larger in the p.V1316A mutant than that of the WT channels with the repolarization potentials between –20 and –70 mV. *; *p* < 0.05. (D) The inverses of the time constants for the decay phase of resurgent Na^+^ currents (the same data as that in Fig 5, *n* = 10) was plotted against the voltage in semi-logarithmic scales for the WT and p.V1316A mutant channels. The lines are linear regression fits of the form: 1/tau_(V)_ = 0.06×exp(–0.81V/25) ms^-1^ for WT channel and 1/tau_(V)_ = 0.04×exp(–0.97V/25) ms^-1^ for p.V1316A mutant channel, respectively, where V is the membrane potential in mV. The inverses of time constants of decay kinetics in the resurgent currents are very similar in the WT and p.V1316A mutant channels. (E) The cells were held at –120 mV and stepped to +40 mV for 0.5 ms of depolarization (the activation pulse), and following by repolarization from –80 mV to –20 mV for ~20 ms (the deactivation pulse) in the WT and p.V1316A mutant channels. The tail currents show faster decay kinetics as the deactivating pulse goes more negative. (F) The decay phase of tail currents in part **E** is fitted by mono-exponential functions for different deactivating potentials in the WT and p.V1316A mutant channels. The inverses of time constants of decaying phase in tail currents are plotted against voltage in semi-logarithmic scales for WT and p.V1316A mutant channels (*n* = 10). The lines are linear regression fitted of the form: 1/tau_(V)_ = 0.97×exp(–0.72V/25) ms^-1^ for WT channel, and 1/tau_(V)_ = 1.25×exp(–0.71V/25) ms^-1^ for p.V1316A mutant channel, respectively, where V is the membrane potential in mV. Note that the inverses of time constants of decay kinetic phases in the tail currents are very similar in the WT and p.V1316A mutant channels. Individual data is shown in the file of S1 Data.

In addition to amplitude, we also investigated the changes in the kinetics of resurgent currents caused by the p.V1316A mutation (Fig 7A and 7B). The kinetics of resurgent current decay at –60 mV remain unaltered over a wide range of prepulse depolarization (i.e., +40~+160 mV). In contrast, the time to peak resurgent currents is significantly longer in the p.V1316A mutant than in the WT channel (Fig 7B and 7C). This could be consistent with the increased sustained and resurgent currents in the p.V1316A mutant channel, which may be chiefly and straightforwardly explained by an acceleration transition from the inactivation to open state (i.e., “destabilized” inactivated states). Consistent with the findings in Fig 5, the “re-opening” of the inactivated channel then very likely involves conformational changes of the domain III, S4–S5 linker where V1316 is located (see Discussion). Fig 7D further shows that the kinetics of decay of the resurgent currents are very similarly accelerated by membrane hyperpolarization in both WT and p.V1316A mutant channels. Interestingly, the kinetics of the decay phase of the resurgent currents are always markedly (~10-fold) slower than that of the deactivating tails of the transient currents (Fig 7D–7F). Given the very short time to peak resurgent currents (<~1 ms in the WT channel, <~2 ms in the p.V1316A mutant channel, Fig 7C), this finding is hard to envisage with the conventional model, which has only one open state. This finding further strengthens the views that there are two distinct open states responsible for the transient and resurgent currents, respectively, and that the exit rates from the open state responsible for the resurgent currents remain unchanged by the p.V1316A mutation (see Discussion).

### Smaller Resurgent Na^+^ Currents with Lengthening of the Depolarization Prepulse

**Fig 8:**
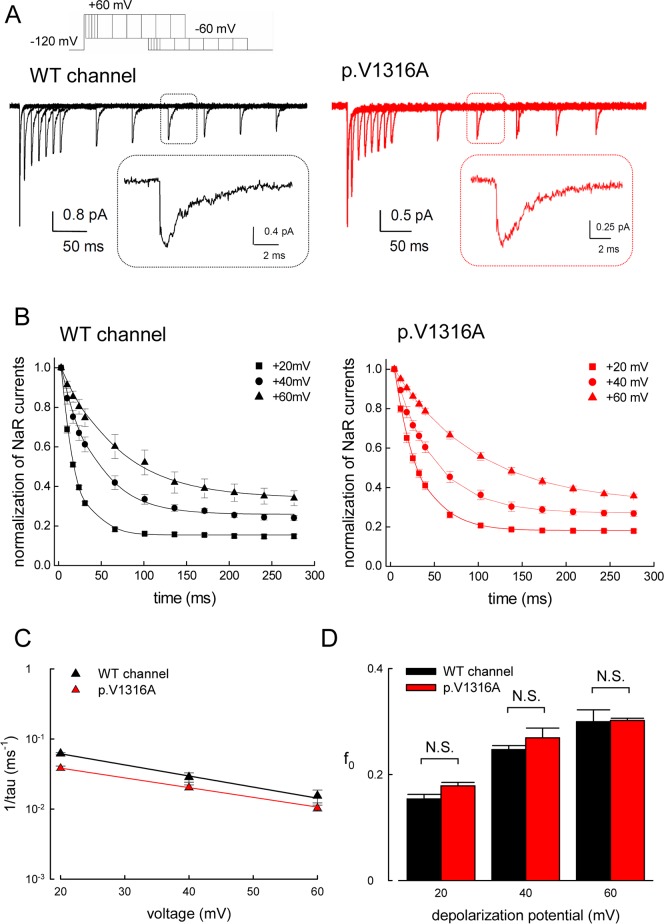
Smaller resurgent Na^+^ currents with lengthening of the depolarization prepulse. (A) The cell was first held at –120 mV, and then stepped to a gradually lengthened depolarization prepulse at +60 mV before stepped to –60 mV for 280 ms to document the resurgent currents. Note that the resurgent Na^+^ currents get smaller with lengthening of the prepulse in the WT and p.V1316A mutant channels. Sample resurgent Na^+^ currents magnified in time scale are shown in inset figures. (B) The normalized amplitude of resurgent Na^+^ currents (normalized to the first current in each series) is plotted against the length of the prepulse (to +20, +40, and +60 mV, the protocols were the same as that in part **A**). The lines are fits to the data points of the form: normalized resurgent currents = (1–f_o_)× exp(–(x–3)/*τ*)+ f_o_, where x is the prepulse length in ms. *τ* and f_o_ are 16.34 ms and 0.15 for +20 mV, 37.6 ms and 0.26 for +40 mV, and 64.88 ms and 0.34 for +60 mV prepulse, respectively, in the WT channel (each *n* = 7). On the other hand, *τ* and f_o_ are 28.73 ms and 0.18 for +20 mV, 44.41 ms and 0.27 for +40 mV, and 98.84 ms and 0.31 for +60 mV prepulse, respectively, in the p.V1316A mutant channel (each *n* = 7). (C) The inverses of time constants in part **B** are plotted against the prepulse voltage in semi-logarithmic scale. The data are fitted with the following equation 1/tau_(V)_ = 0.13×exp(–0.9V/25) ms^–1^ for the WT channel, and 1/tau_(V)_ = 0.07×exp(–0.8V/25) ms^–1^ for the p.V1316A mutant channel, respectively, where V is the prepulse potential in mV. (D) The f_o_ (the residual resurgent Na^+^ currents) in part **B** are plotted against different depolarization potentials (e.g., +20, +40, and +60 mV) in the WT and p.V1316A mutant channels. Note that there is no significant difference between the WT and p.V1316A mutant channels at each voltage (*n* = 7; N.S., no statistically significant difference). Individual data is shown in the file of S1 Data.

In view of the differences in the activation curves of the resurgent currents between 3 ms and 30 ms depolarization prepulses (Fig 6B), we examined the changes in the resurgent currents with a gradually lengthened prepulse. It is intriguing that the resurgent current gets smaller with lengthening of the depolarization prepulse in the WT and p.V1316A mutant channels (Fig 8A and 8B). These findings indicate that with depolarization prepulse 3 ms in length, the WT and p.V1316A mutant channels have not yet reached a steady-state distribution. Moreover, it is easy to reach the new “resurgent” open and the corresponding inactivated states in 3 ms, but subsequent distribution of the channel protein favors the conventional inactivated state, which does not have to go through an open state to deactivate during the following repolarization (see Discussion). The voltage dependence of the kinetics and the “steady-state” relative residual resurgent currents are quite the same in the WT and p.V1316A mutant channels, although the absolute speed of decay is ~2–3-fold slower in the p.V1316A mutant than in the WT channel (Fig 8C and 8D).

## Discussion

### A New Open State Responsible for the Genesis of Resurgent Na^+^ Currents

**Fig 9:**
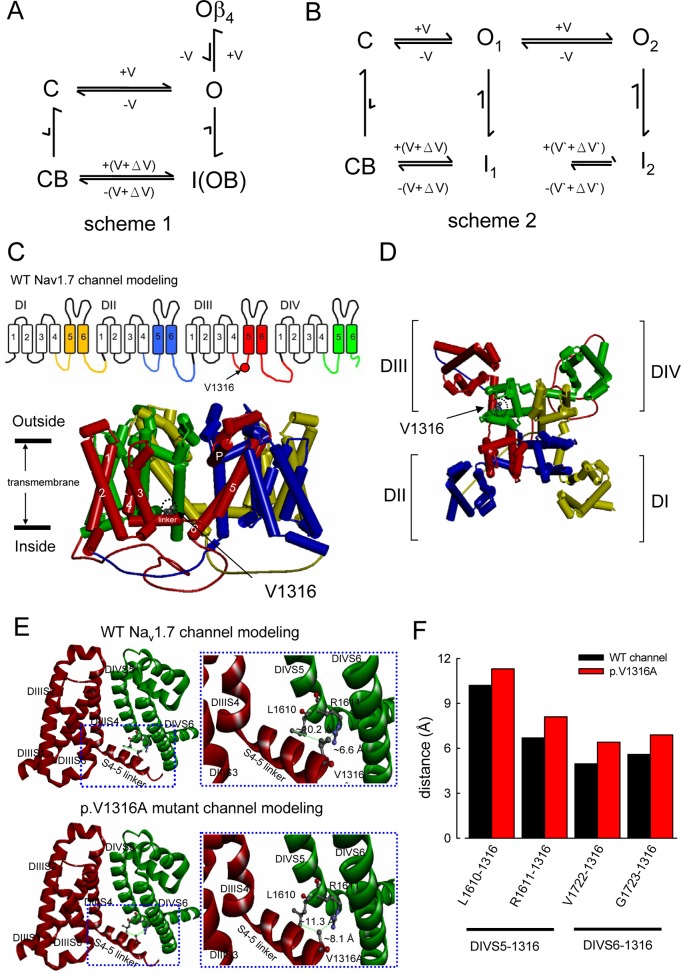
Gating schemes of the Na_v_1.7 channel and the genesis of the resurgent currents. (A) The conventional view of the action of the Na_v_β4 peptide on Na^+^ channel (scheme 1). C and O are the closed and open states, respectively. +V and–V (or +V’ and–V’) denote voltage-dependent rates accelerated by depolarization and hyperpolarization, respectively. The inactivation state (I) is virtually an open but blocked state (OB), the making of which is coupled to channel activation (C to O to I) because of the essentially negligible (the very slow) C to CB transition. The “+(V+ΔV)” and “–(V+ΔV)” indicate that the voltage dependence of the transitions between state OB (i.e., state I) and CB would be shifted by ΔV for the binding energy of the inactivating peptide. In the absence of Na_v_β4 peptide, the recovery of the inactivated channel would primarily take the I to CB to C route due to the very slow I to O transition, and thus result in no currents during the recovery process. The Na_v_β4 peptide is a pore blocker that competes with the inactivating peptide for the open channel pore. The Oβ_4_ is therefore also a blocked state allowing no current passage, but could only go through the open state to the closed state during hyperpolarization. The resurgent currents are thus generated at the repolarization phase following a depolarization. The size of the arrows in the scheme roughly denotes the relative rate of the transition. The rate from O to Oβ_4_ cannot be negligible compared to that from O to I for discernible resurgent currents to occur. (B) A new model for the genesis of resurgent currents (scheme 2). O_1_, O_2_, I_1_, and I_2_ are the first and second open and inactivated states, respectively. The Na_v_β4 peptide is primarily a gating modifier giving rise to the new gating states O_2_ and I_2_, rather than a pore blocker competing with the inactivating peptide. Upon a strong-enough depolarization, a sizable amount of channels in state O_1_ would quickly move to state O_2_ and then inactivated (state I_2_) because the O_1_ to O_2_ transition is very fast and no longer negligible compared to the O_1_ to I_1_ transition. The I_2_ to I_1_ transition is relatively slow so that the recovery from I_2_ upon repolarization would have a significant chance to take the I_2_ to O_2_ to O_1_ to C route and give rise to discernible resurgent currents. Moreover, although the steady-state occupancy in general favors I_1_ over I_2_ (the steady-state I_1_/I_2_ ratio is weakly voltage-dependent and is larger at +20 than at +60 mV, Figs 8 and 10), the redistribution from I_2_ to I_1_ is relatively slow and would take ~10 ms or longer to accomplish (Figs 8B and 10D). The recovery from state I_1_, on the other hand, would primarily go through the CB to C route because of the extremely slow rate from I_1_ to O_1_, and thus allows no resurgent currents. The resurgent current therefore is a transitional phenomenon, not only because state O_2_ is itself transient, but also because the inactivated state giving rise to O_2_ during recovery (i.e., I_2_) is also mostly transient (rather than an “end” state) at a fixed membrane depolarization. (C) The homology model is constructed based on X-ray crystal structures of *Arcobacter butzleri* voltage-gated Na^+^ channel (Na_v_Ab) of a closed-pore conformation using Discovery studio V3.0 software (see Material and Methods for more detail). Side view of the homology model of WT Na_v_1.7 channel shows the transmembrane part of the four domains. Domains I, II, III, and IV are colored yellow, blue, red, and green, respectively. Transmembrane segments (S1–S6) of domain III are highlighted. The side chain of V1316 in the S3–S4 linker of domain III is indicated in the ball and stick model. (D) A regional view of the homology model of WT Na_v_1.7 channel from the extracellular side of the pore. (E) Two domains (domain III and IV) of the homology model of WT Na_v_1.7 and p.V1316A mutant channels are shown in the ribbon presentation. The side chains of V1316 in the (S4–S5 linker/D3), L1610 and R1611 (both in S5/D4) are indicated with sticks and balls of different colors. An enlarged view of the boxed area is shown in the right panel, demonstrating inter-residue distances (from side chain tip to tip) of ~10.2 Å and ~6.6 Å between V1316 and L1610 and between V1316 and R1611, respectively, in the WT Nav1.7 channel. On the other hand, the inter-residue distances (from side chain tip to tip) between p.V1316A and L1610, and p.V1316A and R1611 are ~11.3 Å, ~8.1 Å, respectively, in the p.V1316A mutant channel. (F) A summary plot of the relative (tip to tip) distances between residues V1316 (or p.V1316A) and L1610, R1611, V1722, and G1723 in the homology modeling. Note the evidently increased distances in all cases in the p.V1316A mutant than in the WT channels (see also S2 Fig). Individual data is shown in the file of S1 Data.

**Fig 10:**
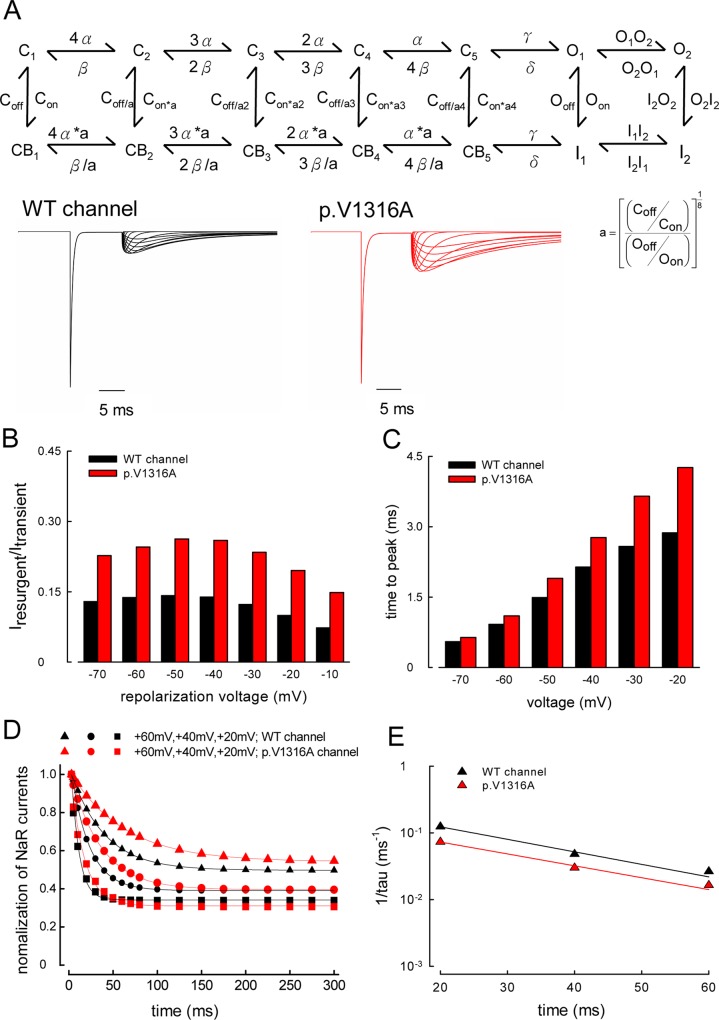
Computational simulations of resurgent Na^+^ currents in the WT and p.V1316A mutant channels. (A) The gating scheme and simulated currents of WT and p.V1316A mutant channels with the same stimulation protocols identical to that in Fig 5A and 5B. The peak of transient currents has been adjusted to be the same in both sets of the simulated currents. There are two open (O_1_ and O_2_) and two corresponding inactivated (I_1_ and I_2_) states. O_2_ and I_2_ are responsible for the genesis of resurgent currents and are significantly existent only in the presence of appropriate gating modifiers such as Na_v_β4 peptide (see Discussion for more molecular and biophysical details). The full set of kinetic parameters is provided in Table 1. In comparison with the WT channels, the main gating changes caused by the p.V1316A mutation include larger rate constants for α, β and I_2_O_2_, and smaller rate constants for O_2_I_2_, I_1_I_2_ and I_2_I_1_. (B) The peak amplitude of the simulated resurgent currents with the same protocols in Fig 5C. (C) The time to peak of resurgent currents was simulated with the same protocols in Fig 7C. (D) The decay of resurgent current with lengthening of the depolarization prepulse is simulated with the same experimental protocol in Fig 8. The lines are fits to the data points of the form: normalized resurgent currents = (1–f_o_)× exp(–(x–3)/τ)+ f_o_, where x is the prepulse length in ms. τ and f_o_ are ~8.1 ms and 0.34 for +20 mV, ~20.8 ms and 0.39 for +40 mV, and ~37.8 ms and 0.49 for +60 mV prepulse, respectively, in the WT channel. On the other hand, τ and f_o_ are ~13.6 ms and 0.31 for +20 mV, ~33.3 ms and 0.39 for +40 mV, and ~60.9 ms and 0.54 for +60 mV prepulse, respectively, in the p.V1316A mutant channel. (E) The inverses of time constants in part **D** are plotted against the prepulse voltage in semi-logarithmic scale. The data are fitted with the following equation 1/tau_(V)_ = 0.29×exp(–1.1V/25) ms^–1^ for the WT channel, and 1/tau_(V)_ = 0.17×exp(–1.03V/25) ms^–1^ for the p.V1316A mutant channel, respectively, where V is the prepulse potential in mV. Individual data is shown in the file of S1 Data.

We have seen that in the Na_v_1.7 channel, resurgent currents are discernible only in the presence of the Na_v_β4 peptide (Fig 2). According to the conventional model of genesis of resurgent currents [27–30], this is ascribable to the competition between the Na_v_β4 peptide and the inactivating peptide for the open channel pore (Fig 9A, scheme 1). However, we have also seen that amplitude of the sustained currents and the kinetics of decay of the transient currents (at prepulses to +40 mV where resurgent currents could be effectively generated) remain unchanged with the addition of Na_v_β4 peptide (Fig 2B and 2C). In the p.V1316A mutant channel, the addition of Na_v_β4 peptide even significantly slows the decay of the transient currents (Fig 4E), although the subsequent resurgent currents are larger than that in the wild-type channel (Fig 5C). Similar findings can be seen in the Na_v_1.1 channel [33], whereas acceleration of the decay phase of the transient current, to our knowledge, has never been reported. These findings are incompatible with the conventional model, which should show an increase in the rate of macroscopic “inactivation” (the rate of decay of the transient currents in the prepulse) and a decrease of sustained currents with the addition of an effective competitive blocker to the system. This would be especially so considering that the resurgent current always very quickly and effectively reaches its peak amplitude within a prepulse just 3 ms in length (Figs 6 and 8). Quite a few other findings may further argue against the hypothesis that the Na_v_β4 peptide competes with the inactivating peptide for the same open state to generate the resurgent currents: (1) decay of resurgent currents with lengthening of the depolarizing prepulse and a time constant from a few to few tens of millisecond, which is shorter at less depolarized prepulses (Figs 8B, 8C, 10D and 10E); (2) the very much positive shift activation curve of the resurgent current but effective negative shift of the activation/inactivation curve by the Na_v_β4 peptide (Figs 3 and 6); and (3) the very short time to peak resurgent currents but evidently much slower decay rates of the resurgent currents than the tail currents at the same negative voltages (Fig 7D and 7F). In addition, the major findings with the p.V1316A mutation, such as the concomitant increase of resurgent and sustained currents, the slowed decay (inactivation) phase of transient currents only in the presence but not absence of Na_v_β4 peptide, and the lengthened time to peak but essentially unaltered decay kinetics as well as the (quite positively located) activation curve of resurgent currents (Figs 4D, 4E, 6C, 7B–7D, 10A and 10B and S1 Fig), would be quite difficult to envisage with the conventional model. We therefore propose that there are at least two distinct open states (and corresponding inactivated states) of the Na_v_1.7 channel, each responsible for the transient (O_1_) and resurgent (O_2_) currents, respectively (Figs 6, 9B and S1). Significant occupancy of O_2_ and I_2_ is possible only in the presence of the Na_v_β4 peptide. In this regard, the Na_v_β4 peptide is chiefly a gating modifier, which induces new gating conformations rather than being a pore blocker that competes with the inactivating peptide (Fig 10 and S1 Fig). If I_2_ is chiefly ascribable to a conformational change in the pore rather than a simplistic pore-blocking phenomenon, it might be interesting to re-visit the role of conformational changes in the conduction pathway in the genesis of I_1_ (versus a pore-blocking phenomenon based on the ball and chain or hinged-lid models), especially considering the decisive and delicate control of Na^+^ channel inactivation and conformation of the conduction pathway (i.e., different single channel conductances) by the movement or positioning of S4 in domain IV (S4/D4) [34–37].

**Table 1:** Kinetic parameters for the wild type (WT) and p.V1316A mutant Na_v_1.7 channels with and without Na_v_β4 peptide.

	WT channel without Na_v_β4 peptide		WT channel with Na_v_β4 peptide		p.V1316A mutant channel with Na_v_β4 peptide	
	*k*_0_ (ms^-1^)	*k*_1_ (mV^-1^)	*k*_0_ (ms^-1^)	*k*_1_ (mV^-1^)	*k*_0_ (ms^-1^)	*k*_1_ (mV^-1^)
α	40	0.08	100	0.08	500	0.08
β	0.55	–0.01	0.55	–0.01	4	–0.01
γ	40	0.04	40	0.04	40	0.04
δ	0.2	–0.04	0.2	–0.04	0.2	–0.04
C_off_	0.5		0.5		0.5	
C_on_	0.1		0.1		0.1	
O_off_ (I_1_O_1_)	0.004		0.004		0.004	
O_on_ (O_1_I_1_)	2.5		2.5		2.5	
O_1_O_2_	N.A.		0.12	0.057	0.12	0.057
O_2_O_1_	N.A.		0.001	–0.095	0.001	–0.095
I_2_O_2_	N.A.		0.1	–0.03	0.13	–0.03
O_2_I_2_	N.A.		0.5	0.04	0.3	0.04
I_1_I_2_	N.A.		0.002	0.01	0.001	0.01
I_2_I_1_	N.A.		0.2	–0.038	0.12	–0.038

Voltage-dependent rates are in general expressed as *k*_(v)_ = *k*_(0)_×exp(*k*_1_×V), where *k*_(v)_ stands for O_1_O_2_, O_2_O_1_, O_2_I_2_, I_2_O_2_, I_1_I_2_, and I_2_I_1_ at a designated membrane potential V (in mV). N.A., non-applicable.

### Much Faster Transition from O_1_ to O_2_ than from I_2_ to I_1_ in the Presence of Gating Modifier Na_v_β4 Peptide

The activation curve of resurgent currents is markedly shifted toward the positive voltages compared with the transient currents (~60 mV difference in V_h_) but has a much shallower slope (slope factors, ~30–40 versus ~8, Fig 6C). It seems that only in the presence of appropriate gating modifiers, such as the Na_v_β4 peptide, may gating voltage sensors move further from their position responsible for O_1_/I_1_ to O_2_/I_2_, this transition is characterized by an apparent transfer of 0.7–0.8 equivalent gating charge and a large non-electrical work, considering the much shallower slope and positively shifted V_h_ of the activation curve of resurgent currents than that of the transient currents. Most intriguingly, the resurgent currents always reach it peak within 3 ms of prepulse initiation, and then decays with a slightly voltage-dependent time constant of 12–40 ms in the wild-type channel (Fig 8). This cannot be explained with the conventional model, where the binding of the competing inactivating and resurgent peptides should be both very fast (the decay time constant of the macroscopic transient currents is only ~0.5 ms, Fig 2B) and thus very fast kinetics approaching the steady-state distribution of the (blocker-) bound states. On the other hand, this finding could be easily envisioned with a very fast transition from O_1_ to O_2_ during prepulse depolarization based on the new model (scheme 2, Fig 9B). In view of the very short time constant of decay of the macroscopic transient currents (~0.5 ms, Fig 2B), very fast O_1_ to O_2_ is actually reasonable or even mandatory for resurgent currents genesis so that O_1_ to O_2_ transition is not negligible compared with O_1_ to I_1_ transition for a channel protein at state O_1_. Therefore, strong rather than weak depolarization (e.g., +40 versus 0 mV) is necessary for sufficient acceleration of the weakly voltage-dependent O_1_ to O_2_ transition to produce discernible resurgent currents. Thus, O_2_ and I_2_ are transitional states in the “detour” route (from O_1_ and finally to I_1_) favored by strong depolarization [34]. Consistently, the “steady-state” relative resurgent currents (f_o_; Figs 8 and 10) are larger with stronger prepulse depolarization (e.g., +60> +40> +20 mV). Under such circumstances, the decrease of peak resurgent currents with lengthening of the prepulse depolarization would signal redistribution of the channel back to O_1_/I_1_ conformations from O_2_/I_2_ conformations during the prepulse (Figs 8 and 10D). The relatively slow kinetics of this redistribution suggests that at depolarizing prepulses, a substantial number of channels first go from state O_1_ to O_2_, then to I_2_, and finally to I_1_, with I_2_ to I_1_ being the slowest or rate-limiting step, requiring at least tens of milliseconds to complete (Figs 9B and 10A). The unaltered sustained currents in Fig 2C by the Na_v_β4 peptide could be straightforwardly represented by unaltered distributions between O_1_ and I_1_. The meanings of all of the major findings could be numerically recapitulated or illustrated by computer simulation based on the two-open-state model (Figs 10 and S1), further substantiating that there are two distinct open and corresponding fast inactivated states underlying the genesis of transient and resurgent Na^+^ currents in the human Na_v_1.7 channel.

### An Increase in Sustained and Resurgent Na^+^ Currents by the p.V1316A Mutation

The p.V1316A mutant Na_v_1.7 channel is characterized by a marked increase in both sustained and resurgent Na^+^ currents (Figs 4D, 5C, 10B and S1). This is the first quantitative report for a specific IEM–causing mutation which concomitantly increases both sustained and resurgent currents. Moreover, the increase in both currents is evident over a wide range of membrane voltages (Figs 4 and 5), suggesting an effective promotion of repetitive discharges by p.V1316A mutation in different firing patterns (e.g., burst discharges with a plateau or not). Based on scheme 2 (Fig 9B), both increased sustained and resurgent currents can be chiefly and straightforwardly explained by an accelerated transition from the new inactivated to new open state (increased I_2_ to O_2_; i.e., a “destabilized” inactivated state). Also, the increase in sustained currents may be envisioned by the shift of activation and inactivation curves in opposite directions (Figs 4B, 6C and S1), signaling uncoupling between activation and inactivation by the p.V1316A mutation. These findings would provide imperative insight into the molecular pathogenesis of IEM, and is well in line with the findings that the S4–S5 linker in domain III (S4-5 linker/D3) interacts with S6 and/or S4–S5 linker in domain IV (S6/D4 or S4-5 linker/D4) to make the most important structural element relaying movement of voltage sensors to conformational changes in the activation and inactivation gates at the internal pore mouth, and thus critically contributes to Na^+^ channel gating including activation/inactivation coupling [14,15,34,38,39]. In this regard, it is especially interesting to note the evident correlation between the voltage-dependent entry kinetics into a subconductance level and the movement of voltage sensor in domain 4 (S4/D4) in an inactivation-deficient Na_v_1.4 channel [34], a very intriguing finding providing a strong support for a critical conformation change in the conduction pathway induced by the movement of S4/D4. A natural extension of such a molecular rationale of Na^+^ channel gating may well underlie the creation of a different open state responsible for the genesis of resurgent currents with 0.7–0.8 more charge transfer or slightly further outward movement of S4/D4 (Fig 6). Consistently, homology modeling of Na_v_1.7 channel shows that the p.V1316A mutation may increase the inter-residue distances between residue p.V1316A of the S4–S5 linker/D3 and the nearby residues (e.g., L1610, R1611, V1722, and G1723) of the S5–6 segments in domain IV (Figs 9E, 9F and S2). Here we do not mean to make any rigorous argument based on these distances from modeling. Instead, it is a demonstration of the possibility that the conformational changes associated with p.V1316A mutation may well involve the pivotal points of channel activation/inactivation, and thus be responsible for inactivation weakening and activation/inactivation uncoupling (Fig 4E).

### The Electrophysiological Pathogenesis of IEM and Therapeutic Implications

IEM and paroxysmal extreme pain disorder (PEPD) are characterized by episodic excruciating regional pain associated with hyperemic swelling (Fig 1), presumably manifestations of sensory (dorsal root ganglia) and sympathetic neuronal hyperexcitability. Electrophysiologically, it has been suggested that IEM mutations tend to make negative shift of the Na_v_1.7 channel activation curve and also slow the macroscopic deactivation kinetics, whereas PEPD mutations tend to make a positive shift of the inactivation curve, slow macroscopic activation kinetics, and generate more persistent as well as resurgent Na_v_1.7 currents [40–42]. In this study, we demonstrated that the p.V1316A mutation, which causes very prominent and typical clinical features of IEM, resulting in not only negative and positive shift of the activation and inactivation curves, respectively, but also evidently larger sustained and resurgent currents. IEM and PEPD thus may be more a clinical classification than a legitimate electrophysiological differentiation. An “appropriate” amount of increased sustained currents might depolarize the neuron to a level not to inactivate too many Na^+^ channels and thus easier for an external stimulus to fire an action potential. Once the first action potential is fired, the subsequent discharges are powered by the resurgent Na^+^ currents. The largest amplitude of resurgent currents with very short preceding depolarization (Figs 8, 10D and 10E), would also these currents especially suitable for the genesis of densely repetitive spikes or “burst-like” discharges [41,43]. The dorsal root ganglia sensory neurons and sympathetic ganglion neurons carrying IEM-causing Na_v_1.7 mutations then could be hyperexcitable, overly responding to an ordinarily innocuous stimulus with excessive bursts of discharges. The episodes of painful attacks of IEM/PEPD therefore carry some of the essential electrophysiological features of epileptic seizures. From a therapeutic point of view, these “peripheral seizures” in IEM/PEPD patients are most different from classical seizures in the general ineffectiveness of conventional anticonvulsants acting on the Na^+^ channel [15,42,44]. We have noted that the gating states responsible for resurgent currents (O_2_ and I_2_ in scheme 2, Figs 9B and 10A) exist only transiently. The anticonvulsants with slow binding rates onto the inactivated Na^+^ channel (e.g., phenytoin, carbamazepine, and lamotrigine [45–47]) therefore may not have enough time to bind to the specific gating states to inhibit resurgent currents. It would be interesting to explore the molecular interaction between different anticonvulsants and the novel “resurgent” gating states (i.e., O_2_ and I_2_) in more detail, and thereby search for more effective pharmacotherapy of IEM and related disorders.

## Materials and Methods

### Laser Doppler Flowmetry

The clinical investigations were approved by the Institutional Review Board (IRB) of National Taiwan University Hospital Ethics Committee (Permit Number: NTUH–REC No. 9461700723). The control subjects for the clinical investigations are six (three female and three male) age-matched individuals without any symptoms and signs in both past and present medical histories. Also, each of the control subjects must have normal results in the nerve conduction velocity study prior to recruitment. The patient and control individuals were allowed to rest quietly in an air-conditioned room (20–25°C, humidity 50%) for at least 20 min before initiating the flowmetry measurement using a laser Doppler flowmeter (PeriFlux, PF3, Perimed, Sweden). The principles underlying laser Doppler flowmetry are described elsewhere [48]. A laser Doppler output signal of more than 90% by flow in the subpapillary vessels was continuously monitored [49]. The signals at baseline, during and after thermal stimulation were recorded. Thermal stimulation was achieved by directly heating with laser probe for 1 min in order to raise the skin temperature to 44°C. Skin blood flow was measured immediately after thermal stimulation and again after a 10 min cooling rest period in the air condition room. All measures were expressed in perfusion unit (P.U.). Theoretically, the flow is determined by the product of blood flow velocity and the number of moving red cell corpuscles within the surface capillaries of the skin. The mean value of the perfusion unit in a defined period was analyzed by Perisoft software (the Perimed analysis program for PeriFlux). Each measurement was repeated six times in both the patient and seven normal age-matched controls. The intra-individual coefficient of variations was ~9.8%. Just prior to the blood flow test, the temperature of the skin was taken using an electric thermometer (Takara, Japan) to measure the temperature at baseline, during, and after thermal stimulation. Mann-Whitney U-test was used for non-parametric comparison among the measurements of the index patient and the control group.

### Molecular Biology and Expression DNA Constructs of Na_v_1.7 Channel

The human Na_v_1.7 cDNA (*SCN9A* gene) was subcloned into pTracer–EF/V5–His vector [15]. The GFP sequence, which is driven by a separate promoter in pTracer vector, allows identification of the transfected cells under fluorescent microscope (Nikon, Inc. Japan). The p.V1316A mutation was made using Quikchange site-directed mutagenesis kit (Stratagene, La Jolla, CA, US), and confirmed by the automatic DNA sequencing (Applied Biosystems, 3730xl DNA, Analyzer Foster, CA, US) [15,50–53].

### Preparation of Cell Line for Transfection

Chinese hamster ovary (CHO–K1) cells were obtained from Food Industry Research and Development Institute (Hsinchu, Taiwan) [15]. CHO–K1 cells were approved by the Institutional Biosafety and Use Committee (IBUC) and conformed to the ethical information guidelines of the National Taiwan University College of Medicine. CHO–K1 cells were cultured under the standard conditions (37°C, 5% CO_2_) in F12–K medium (Thermo Fisher Scientific Inc., US) with 10% fetal bovine serum (FBS, Thermo Fisher Scientific Inc., US) and 1% Penicillin–Streptomycin (Thermo Fisher Scientific Inc., US). The cells were transfected with either wild-type (WT) or p.V1316A mutant cDNA constructs of the human Na_v_1.7 channel using Lipofectamine 3000 (Thermo Fisher Scientific Inc., US) [15]. The cells were then maintained at 37°C, 5% CO_2_ incubator before electrophysiological recordings. For recordings, CHO–K1 cells were treated with 1.0 mg/ml protease type XXIII (Sigma Chemical Co., St Lois, MO) [54], dissociated in F12–K medium with 10% FBS, and plated on glass cover-slips at 37°C, 5% CO_2_ incubator for 30–40 min. The enzyme action was terminated by wash with F12–K medium. Usually the recordings were carried out within 2–3 d following DNA transfection, and the isolated CHO–K1 cells were used within 5 h of preparation.

### Electrophysiological Recordings

Whole-cell patch recordings from CHO–K1 cells expressing WT or p.V1316A mutant channels were conducted at room temperature using the pClamp 6.0 software and an Axopatch 200A amplifier (Axon Instruments, Inc. Sunnyvale, CA, US). Data were filtered at ~10 kHz and digitized at ~50 kHz through Digidata 1200A interface (Axon Instruments, Inc. Sunnyvale, CA, US). The pipettes were pulled from borosilicate glass (Warner Instruments, MA, US) using a horizontal puller (Zeitz Instruments, Inc. Martinsried, Germany) and fired polished (Narishige scientific instruments, Inc, Japan) [50–53]. The pipette resistance was 1–2 MΩ when filled with intracellular solutions containing (in mM): 75 CsCl, 75 CsF, 5 HEPES, 2 CaCl_2_ and 2.5 EGTA, titrate to pH 7.4 by 1 M CsOH. Whole-cell configuration was obtained and giga-seal formed in the extracellular solution containing (in mM): 150 NaCl, 2 MgCl_2_, 2 CaCl_2_, 10 HEPES, titrated to pH7.4 by 1 M NaOH. The cell was lifted from the cover-glass and moved in front of a linear array of pipes, each containing a different extracellular solution. The current sweeps in Tetrodotoxin (TTX, Torcris Cookson, Langford, UK) were used for the subtraction of leak currents and capacities to obtain the TTX-sensitive currents for subsequent analysis [55]. The Na_v_β4 peptide (KKLITFILKKTREK–OH, the C–terminal half of the whole sequence of the Na_v_β4 subunits [56,57]) remains a standard and effective tool for the investigation of resurgent currents in culture cells or even native neurons in most studies, because the entire β4 subunit is much less effective in the induction of resurgent currents for so-far unknown reasons [32,33]. The Na_v_β4 peptide (Genomics Bioscience and Technology Co., Ltd, Taiwan) was dissolved in distilled water to make a 10 mM stock solution and then diluted into intracellular solution for a final concentration of 0.1 mM [42,58]. Data were analyzed using Clmapfit 9.0 (Axon Instruments, Inc. Sunnyvale, CA, US), and Sigmaplot 10.0 software (Systat software, Inc., Germany).

### Construction of the Activation and Inactivation Curves

For current-voltage relationship, cells were hold at –120 mV and stepped to a range of test voltages (–140 to +40 mV in 5 mV increments) for 100 ms. Maximal inward currents were plotted as a function of test voltage to generate the current-voltage plot. We made a regression line of the currents points between +10 and +40 mV in the current-voltage plots of each cell [22,35,36,59–64], while the maximal Na^+^ currents usually appear at a test voltage between 0 and –20 mV. The reversal potential of Na^+^ (V_rev; Na_^+^) was determined by extrapolating the regression line to the transverse axis, and the maximal Na^+^ conductance (G_max_) was given by the product of ΔV (the difference between each voltage and the reversal potential) and the slope of the regression line from each individual cell. The measured currents at each voltage were divided by the maximal currents at each voltage in the same cell to give G/G_max_. The activation data were then fitted with a Boltzmann function: G/G_max_ = 1/(1+exp(V_h_–V)/*k*)), where G_max_ is the maximal Na^+^ conductance, V_h_ is the potential at which activation is half-maximal, V is the test voltage in mV, and *k* is the slope factor [22,35,36,59–64]. For the steady-state fast inactivation curve, we measured the currents at a +10 mV test pulse after 100 ms prepulse at different potentials from a holding potential of –120 mV. The current measured from the test pulse was normalized to the maximal currents (I/I_max_) in the series from the same cell and plotted against the prepulse voltage. The inactivation data are also fitted with a Boltzmann function I/I_max_ = 1/(1+exp(V–V_h_)/*k*)), where I_max_ is the maximal Na^+^ currents, V_h_ is the potential at which inactivation is half-maximal, V is the prepulse potential in mV, and *k* is the slope factor.

### The Human Na_v_1.7 Channel Homology Modeling

The human Na_v_1.7 channel (*SCN9A* gene) homology modeling was built based on the X-ray crystal structure data of voltage-gated Na^+^ channel in *Arcobacter butzleri* (Na_v_Ab; PDB code: 3rvy) [19,65,66]. The human Na_v_1.7 channel sequence was obtained from the UniProt database (Q15858; http://www.uniprot.org/). The homology modeling was performed in a similar way to that described in previous studies [50–53]. Sequences of the four domains (DI–DIV) of the human Na_v_1.7 channel are very different from those of the Na_v_Ab channel [19,65,66]. We first aligned critical S4–S5 linker residues in each domain (D1, V247–R266; D2, F902–M921; D3, G1305–F1334; and D4, Y1591–L1610) with corresponding S4–5 linker residues in the Na_v_Ab channel (D1: A112–L131, D2: A351–S371, D3: A593–S613, and D4: A872–S892) according to BLAST research results. We then constructed multiple alignments of S5 and S6 sequences of the four domains (DI–DIV) of the human Na_v_1.7 and Na_v_Ab channels (S3 Fig). We aligned residues in S5 of each domain (S5: D1, Y356–F376; D2, D933–I943; D3, G1382–Y1402; and D4, L1610–L1630), and S6 of each domain (S6: D1, C725–V746; D2, I1169–M1190; D3, Y1430–F1441; and D4, S1715–I1736) with corresponding S5–6 residues in the Na_v_Ab channel (S5: S132–F152, and S6: F201–M222) according to BLAST research results. The crystal structure of the Na_v_Ab template sequence that showed higher score in the homologous sequence alignment was chosen as the structure conserved region (SCR) [67]. The identity of S5–6 sequences in each domain between human Na_v_1.7 and Na_v_Ab channels is greater than 35%, with the root mean square (r.m.s.) of the backbone 0.01 Å based on a model including only crystallized proteins [68]. Particularly, full sequences of the three interdomain linkers of the human Na_v_1.7 channel (DI–DII linker, C740–P789; DII–DIII linker, M1190–Y1239; and DIII–DIV linker, D1447–V1519) were inserted into corresponding positions between different domains (DI–DIV) by homology modeling based on the Na_v_Ab channel structure template. The aligned sequences were then presented to Discovery Studio V3.0 (DS V3.0) client program to generate relative positions and the secondary structures in the vicinity of selected residues of the human Na_v_1.7 channel [50–53]. There were 10 human Na_v_1.7 channel homology modelings [50–53], which were subjected to the steepest descent energy minimization with a tolerance of 100 KJ/mol/nm [69], followed by a second conjugate gradient energy minimization with a tolerance of 10 KJ/mol/nm [69] using Discovery Studio V3.0 (DS V3.0) client program. The conformation with the lowest free energy potential estimated by the Discrete Optimized Protein Energy (DOPE; ~132,970) Score was chosen for further analysis.

### Computational Simulations of the Resurgent Na^+^ Currents

The scheme of sodium channel without Na_v_β4 peptide is based on a previous model [54]. The kinetic parameters were adjusted to fit the experimental data, including activation and inactivation curves of transient currents of WT channels in presence or absence of the Na_v_β4 peptide, and p.V1316A mutant channels in presence of Na_v_β4 peptide, peaks of resurgent currents, time to peak of resurgent currents, changes of resurgent currents with lengthening of the depolarization prepulse, and the activation curve of resurgent currents of both WT and p.V1316A mutant channels. The ionic currents are assumed to have Ohmic relationship, and the reversal potential was set as +100 mV. Euler method was used for numeric integration. The maximal time step was set to be 10^−6^ ms. The computations and graphic constructions were performed with QuB express suite (https://www.qub.buffalo.edu/) [70] and MATLAB R2015 suite (The MathWorks, Inc. US).

### Statistical Analysis

All data are presented as mean ± standard error of the mean (S.E.M.). Statistical significance is assessed using Student’s independent *t* test and accepted at *p* < 0.05 (except for Fig 1 in which Mann-Whitney U-test were applied).

## Supporting Information

S1 Data Supplementary excel files in separate sheets containing the individual data and statistical analyses for Figs 1B, 1C, 2B, 2C, 2D, 3B, 4B, 4C, 4D, 4E, 5C, 6B, 6C, 7A, 7B, 7C, 7D, 7F, 8D, 8C, 8D, 9F, 10B, 10C, 10D, 10E and S1A–S1E. (XLS)

S1 Fig The other biophysical features recapitulated by the simulated currents. (A) The ratios between the sustained and peak currents in the WT and p.V1316A mutant channels are obtained with the same pulse protocol as that in Fig 4. Similar to the experimental findings, simulated sustained currents are increased in p.V1316A mutant channels. (B) The kinetics of the resurgent Na^+^ currents in the WT and p.V1316A mutant channels are obtained with same pulse protocols in Fig 7D. The lines are linear regression fits of the form: 1/tau_(V)_ = 0.005×exp(–1.77V/25) ms^-1^ for WT channel, and 1/tau_(V)_ = 0.003×exp(–2.0V/25) ms^-1^ for p.V1316A mutant channel, respectively, where V is the membrane potential in mV. The simulated time constants of decay phase of resurgent sodium currents are similar between the WT and p.V1316A mutant channels. These findings are compatible with those in Fig 7D. (C) The activation and inactivation curves of the WT channel in the absence and presence of Na_v_β4 peptide. The curves are simulated with the same protocol as in Fig 3. The condition in the absence of Na_v_β4 peptide is simplistically obtained by deletion of states O_2_ and I_2_ from the scheme in Fig 10A with a change in α (see Table 1). The activation and inactivation curves are fitted with Boltzmann functions of the form: 1/(1+exp((V_h_–V)/*k*)), where V is the membrane potential, and V_h_ and *k* are –17.1 mV and 12.8 in the presence, and –10.3 mV and 11.4 in the absence of the Na_v_β4 peptide, respectively. For the inactivation curves, the Vh and *k* are –83.4 mV and –6.8 in the presence, and –73.4 mV and –6.7 in the absence of the Na_v_β4 peptide, respectively. (D) The activation and inactivation curves in the p.V1316A mutant channels are simulated with the same protocol as in the Fig 4 (red points). The activation and inactivation curves are fitted with Boltzmann functions of the form: 1/(1+exp((V_h_–V)/*k*)), where V is the membrane potential, V_h_ and *k* are –21.9 mV and 13.9 for the activation curve, and –79.2 mV and –6.7 for the inactivation curve, respectively (red lines). The solid black lines for the WT channel are taken from part **C** for comparison. (E) The activation curves of resurgent currents are simulated with the same experimental protocols as in Fig 6B (prepulse = 30 ms). The lines are best fits to the data points with a Boltzmann function: 1/(1+exp((V_h_–V)/*k*)), where V is the membrane potential, V_h_ and *k* are ~66.6 mV and ~17.4 for WT resurgent currents (black symbols and line), and ~62.1 mV and ~17.2 for p.V1316A mutant resurgent currents (red symbols and line), respectively. Individual data is shown in the file of S1 Data.
(TIF)

S2 Fig The homology modeling for the WT and p.V1316A mutant Na_v_1.7 channels based on the X-ray crystal structure of the Na_v_Ab channel. The diagram of two domains (domain III and IV) of the homology model of WT Na_v_1.7 and p.V1316A mutant channels are in the ribbon presentation. The side chains of V1316 (in S4–5 linker/D3), V1722 and G1723 (both in S6/D4) are indicated with sticks and balls of different colors. An enlarged view of the boxed area is shown in the right panel, demonstrating inter-residue distances (from side chain tip to tip) of ~4.9 Å and ~5.5 Å between V1316 and V1722, and between V1316 and G1723, respectively, in the WT Na_v_1.7 channel. On the other hand, the inter-residue distances (from side chain tip to tip) between p.V1316A and V1722, and p.V1316A and G1723 are ~6.4 Å, and ~6.9 Å, respectively in the p.V1316A mutant channel. Individual data is shown in the file of S1 Data.
(TIF)

S3 Fig Sequence alignment used in the human Na_v_1.7 channel homology modeling. Amino acid sequence alignment for S5 and S6 segments of four domains (D1–D4) of the human Na_v_1.7 channel. S5 and S6 sequences of each domain are shown for the Na_v_Ab channel. Amino acid numbers of the human Na_v_1.7 and Na_v_Ab channels are shown on both sides. We chose the S5–6 amino acid sequences of four domains of human Na_v_1.7 from UniProt data (Q15858; http://www.uniprot.org/) for alignment with the amino acid sequences of Na_v_Ab channels. We aligned residues in S5 of each domain (S5: D1, Y356–F376; D2, D933–I943; D3, G1382–Y1402; and D4, L1610–L1630), and S6 of each domain (S6: D1, C725–V746; D2, I1169–M1190; D3, Y1430–F1441; and D4, S1715–I1736) with corresponding S5-6 residues in the Na_v_Ab channel (S5: S132–F152, and S6: F201–M222) according to BLAST research results. Aligned sequences were then presented to Discovery Studio V3.0 (DS V3.0) client program to generate relative positions and the secondary structure in the vicinity of selected residues of the human Na_v_1.7 channel [50–53].
(TIF)

## Abbreviations

CIP: congenital insensitivity to pain
DRG: dorsal root ganglia
IEM: inherited erythromelalgia
O/I: open/inactivated
OMIM: online mendelian inheritance in man
PEPD: paroxysmal extreme pain disorder
P.U.: perfusion unit
S.E.M.: standard error of the mean
WT: wild type
