# Supplementary figures and images for: The Biophysical Basis Underlying Gating Changes in the p.V1316A Mutant Nav1.7 Channel and the Molecular Pathogenesis of Inherited Erythromelalgia
Source: PLoS Biol. 2016 Sep 21;14(9):e1002561. doi: 10.1371/journal.pbio.1002561 (PMC5031448; doi:10.1371/journal.pbio.1002561)

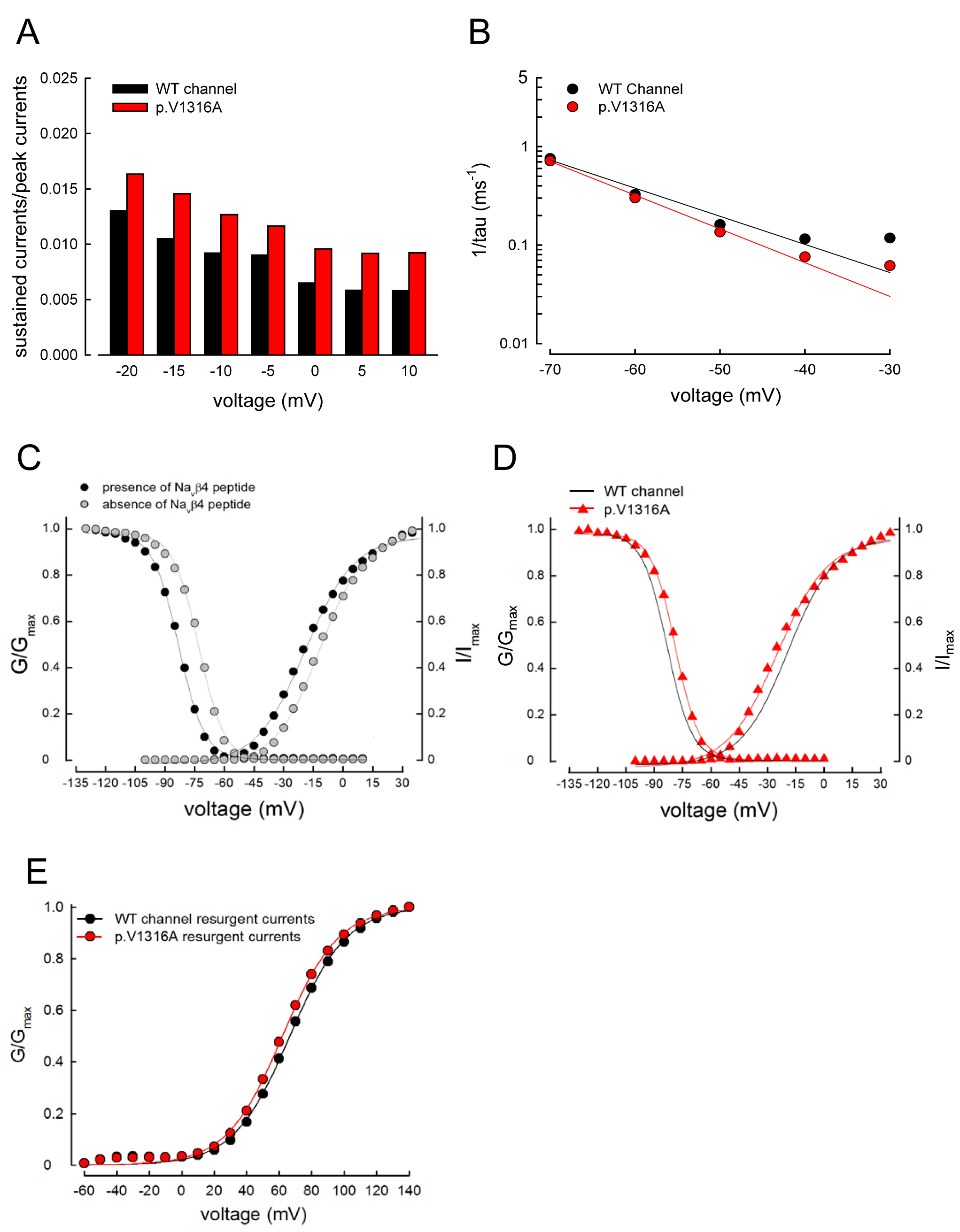

Supplement: S1 Fig — (A) The ratios between the sustained and peak currents in the WT and p.V1316A mutant channels are obtained with the same pulse protocol as that in Fig 4. Similar to the experimental findings, simulated sustained currents are increased in p.V1316A mutant channels. (B) The kinetics of the resurgent Na+ currents in the WT and p.V1316A mutant channels are obtained with same pulse protocols in Fig 7D. The lines are linear regression fits of the form: 1/tau(V) = 0.005×exp(–1.77V/25) ms-1 for WT channel, and 1/tau(V) = 0.003×exp(–2.0V/25) ms-1 for p.V1316A mutant channel, respectively, where V is the membrane potential in mV. The simulated time constants of decay phase of resurgent sodium currents are similar between the WT and p.V1316A mutant channels. These findings are compatible with those in Fig 7D. (C) The activation and inactivation curves of the WT channel in the absence and presence of Navβ4 peptide. The curves are simulated with the same protocol as in Fig 3. The condition in the absence of Navβ4 peptide is simplistically obtained by deletion of states O2 and I2 from the scheme in Fig 10A with a change in α (see Table 1). The activation and inactivation curves are fitted with Boltzmann functions of the form: 1/(1+exp((Vh–V)/k)), where V is the membrane potential, and Vh and k are –17.1 mV and 12.8 in the presence, and –10.3 mV and 11.4 in the absence of the Navβ4 peptide, respectively. For the inactivation curves, the Vh and k are –83.4 mV and –6.8 in the presence, and –73.4 mV and –6.7 in the absence of the Navβ4 peptide, respectively. (D) The activation and inactivation curves in the p.V1316A mutant channels are simulated with the same protocol as in the Fig 4 (red points). The activation and inactivation curves are fitted with Boltzmann functions of the form: 1/(1+exp((Vh–V)/k)), where V is the membrane potential, Vh and k are –21.9 mV and 13.9 for the activation curve, and –79.2 mV and –6.7 for the inactivation curve, respectively (red lines). The solid black [file pbio.1002561.s002.tif]

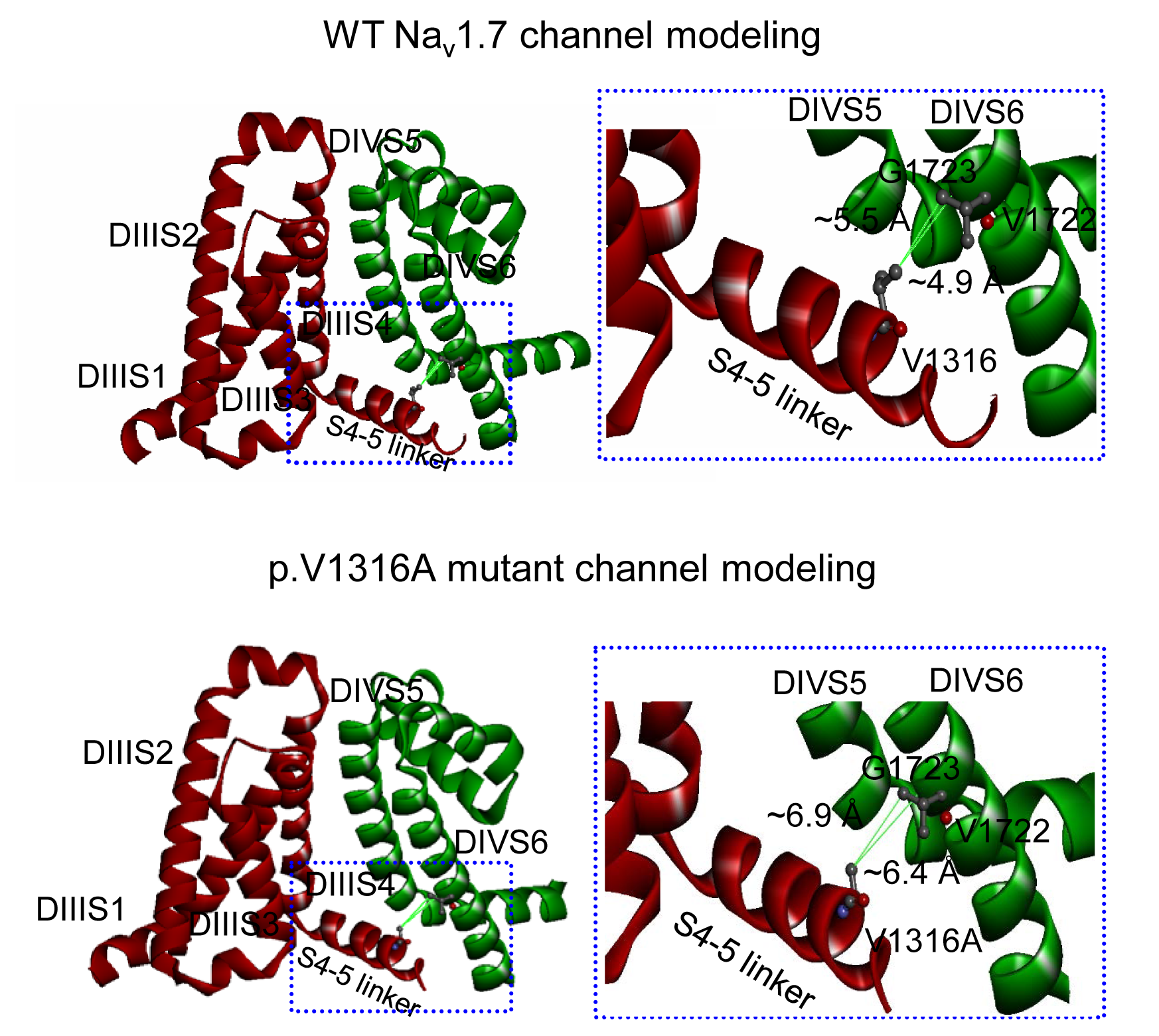

Supplement: S2 Fig — The diagram of two domains (domain III and IV) of the homology model of WT Nav1.7 and p.V1316A mutant channels are in the ribbon presentation. The side chains of V1316 (in S4–5 linker/D3), V1722 and G1723 (both in S6/D4) are indicated with sticks and balls of different colors. An enlarged view of the boxed area is shown in the right panel, demonstrating inter-residue distances (from side chain tip to tip) of ~4.9 Å and ~5.5 Å between V1316 and V1722, and between V1316 and G1723, respectively, in the WT Nav1.7 channel. On the other hand, the inter-residue distances (from side chain tip to tip) between p.V1316A and V1722, and p.V1316A and G1723 are ~6.4 Å, and ~6.9 Å, respectively in the p.V1316A mutant channel. Individual data is shown in the file of S1 Data. (TIF) [file pbio.1002561.s003.tif]

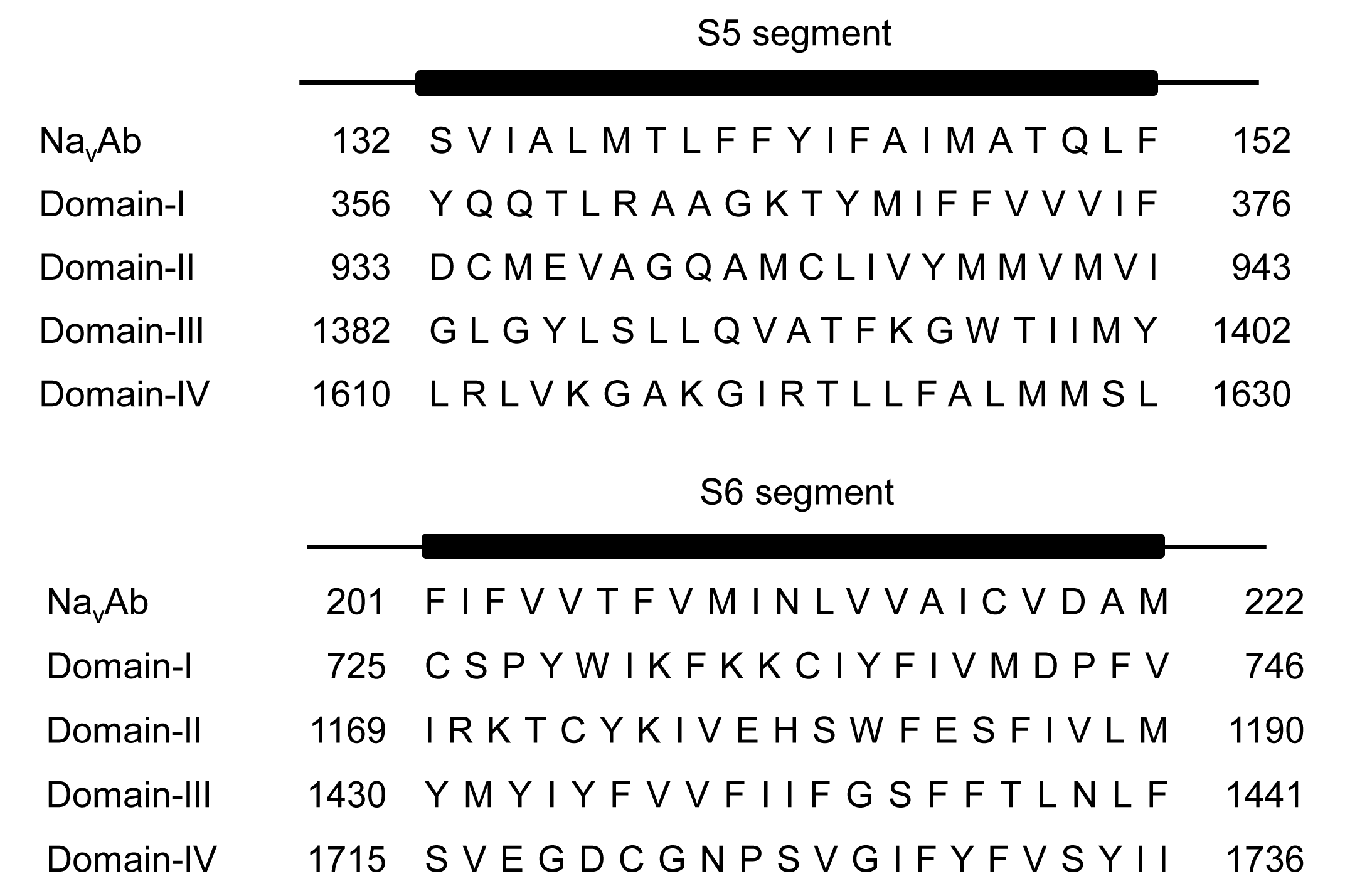

Supplement: S3 Fig — Amino acid sequence alignment for S5 and S6 segments of four domains (D1–D4) of the human Nav1.7 channel. S5 and S6 sequences of each domain are shown for the NavAb channel. Amino acid numbers of the human Nav1.7 and NavAb channels are shown on both sides. We chose the S5–6 amino acid sequences of four domains of human Nav1.7 from UniProt data (Q15858; http://www.uniprot.org/) for alignment with the amino acid sequences of NavAb channels. We aligned residues in S5 of each domain (S5: D1, Y356–F376; D2, D933–I943; D3, G1382–Y1402; and D4, L1610–L1630), and S6 of each domain (S6: D1, C725–V746; D2, I1169–M1190; D3, Y1430–F1441; and D4, S1715–I1736) with corresponding S5-6 residues in the NavAb channel (S5: S132–F152, and S6: F201–M222) according to BLAST research results. Aligned sequences were then presented to Discovery Studio V3.0 (DS V3.0) client program to generate relative positions and the secondary structure in the vicinity of selected residues of the human Nav1.7 channel [50–53]. (TIF) [file pbio.1002561.s004.tif]
